# *Dbx1* is a dorsal midbrain-specific determinant of GABAergic neuron fate and regulates differentiation of the dorsal midbrain into the inferior and superior colliculi

**DOI:** 10.3389/fcell.2024.1336308

**Published:** 2024-01-26

**Authors:** Hong-Nhung Tran, Quy-Hoai Nguyen, Yongsu Jeong

**Affiliations:** Department of Genetics and Biotechnology, College of Life Sciences, Graduate School of Biotechnology, Kyung Hee University, Yongin, Gyeonggi, Republic of Korea

**Keywords:** *Dbx1*, GABAergic neuron, inferior colliculus, superior colliculus, *Tcf7l2*, dorsal midbrain

## Abstract

The mechanism underlying the differentiation of the dorsal midbrain into two morphologically and functionally distinct compartments, the inferior colliculus (IC) and superior colliculus (SC), which process auditory and visual information, respectively, remains largely unexplored. By using null and conditional alleles, we uncover the roles of a homeodomain transcription factor *Dbx1* in the regulation of IC and SC differentiation. We show that *Dbx1* regulates GABAergic neuron development in the dorsal midbrain. In the absence of *Dbx1* function, the dorsal-most m1-m2 progenitor domains in the midbrain fail to activate GABAergic neuron-specific gene expression and instead switch to a glutamatergic phenotype. These results identify *Dbx1* as a dorsal midbrain-specific GABAergic determinant that regulates the selector genes, *Helt*, *Gata2*, and *Tal2*. Furthermore, we demonstrate that maturation of the dorsal midbrain into the IC and SC is dependent on *Dbx1*. Null mutation of *Dbx1* impairs the identity and fate of IC and SC neurons. Surprisingly, *Dbx1* is required for preventing IC into SC fate switch and thus *Dbx1*-deficient IC neurons undergo acquisition of SC identity. Conditional inactivation of *Dbx1* at late developmental phase leads to alteration in the identity and fate of the IC, but not the SC. These results suggest that SC differentiation is dependent on the early function of *Dbx1*, and that the IC requires the prolonged action for its normal formation. Furthermore, we uncover that *Tcf7l2* acts downstream of *Dbx1* selectively to promote IC differentiation. Altogether, our study identifies a molecular mechanism underlying spatial and temporal control of dorsal midbrain development.

## 1 Introduction

Early growth and patterning of the dorsal midbrain are tightly linked to the key signaling molecules of the isthmic organizer (Shamim et al., 1999; Joyner et al., 2000; Wurst and Bally-Cuif, 2001; Chi et al., 2003). FGF signaling functions in a dose- and time-dependent manner to control growth, specification and survival of the dorsal midbrain (Chi et al., 2003; Basson et al., 2008; Dee et al., 2016). *Engrailed*, *Pax* and *Meis2* play differential roles in forming the dorsal midbrain (Urbánek et al., 1994; Matsunaga et al., 2001; Sgaier et al., 2007; Agoston and Schulte, 2009), and *Otx2* contributes to the control of multiple aspects of dorsal midbrain development (Broccoli et al., 1999; Puelles et al., 2003; Di Giovannantonio et al., 2014). Although previous works have greatly broadened our understanding of the early phase of dorsal midbrain development, the molecular basis underlying maturation of the dorsal midbrain at the late embryonic and postnatal stages remains unexplored.

Following the induction of the midbrain during early embryogenesis, the territories of midbrain progenitors are specified into the roof plate, alar plate, basal plate and floor plate along the dorso-ventral axis by Shh and Bmp/Wnt signals (Zervas et al., 2005; Puelles, 2007; Dessaud et al., 2008; Le Dréau and Martí, 2012). These midbrain progenitors are further assigned into seven subdivisions (m1-m7) with specific combinations of gene expression codes (Nakatani et al., 2007; Kala et al., 2009; Lahti et al., 2013). The most dorsal midbrain neuronal populations derived from m1-m2 domains are clustered in the superior colliculus (SC) and inferior colliculus (IC) along the anteroposterior axis. The SC forms its stereotypical laminated structure and differentiates into a number of layers that can be grouped into the superficial layers receiving inputs related to vision and the deeper layers responding to nonvisual multiple modalities (McLaughlin and O’Leary, 2005). The IC located caudal to the SC functions as an acoustic integration center, and develops into the central nucleus (core region) receiving auditory inputs and the lateral and dorsal cortex (shell region) receiving nonauditory projections (Oliver and Morest, 1984; Cant and Benson, 2006). Despite the functional significance of the IC and SC, how the dorsal midbrain differentiates into these two distinct compartments is largely unknown.

Distinct subtypes of GABAergic neurons are formed at different locations and times during midbrain development. GABAergic neurogenesis can be detected first in the ventral midbrain, where GABAergic neuronal markers are expressed at E10.5∼E11.5. By contrast, in the dorsal midbrain, GABAergic neurogenesis begins 1 day later and persists longer (Achim et al., 2012). Basic HLH factors *Helt* (also known as *Megane* or *Heslike*), *Ascl1*, *Tal2* and zinc finger protein *Gata2* have been shown to be indispensable for specifying GABAergic identity in the midbrain (Guimera et al., 2006; Nakatani et al., 2007; Kala et al., 2009; Peltopuro et al., 2010; Achim et al., 2013; Wende et al., 2015). These observations suggest that GABAergic neuronal differentiation requires different dorsoventral molecular mechanisms. However, region-specific determinants or selector genes that govern genetic program remain to be determined.

Recently, we showed that a homeodomain transcription factor *Dbx1* (Developing brain homeobox 1) functions as a key regulator that controls genetic programs for postnatal survival of the IC (Tran et al., 2023). Loss of *Dbx1* causes apoptotic cell death by upregulating c-Jun and pro-apoptotic BH3 only factors and leads to specific loss of the IC. Furthermore, we found that the function of *Dbx1* as a survival factor is mediated by *Tcf7l2* and *Ap-2δ* transcription factors. In the present study, we assess the role of *Dbx1* in regulating developmental acquisition of dorsal midbrain neuron identity. We show that *Dbx1* functions as a dorsal midbrain-specific GABAergic determinant by regulating the selector genes, *Helt*, *Gata2*, and *Tal2*. Furthermore, we uncover that *Dbx1* is required to partition the dorsal midbrain into the IC and SC.

## 2 Materials and methods

### 2.1 Ethics and mouse lines

All animal procedures were carried out in accordance with the guidelines and protocols approved by the Kyung Hee University Institutional Animal Care and Use Committee. The generation of *Dbx1* *^loxp/loxp^* (JAX028223), *Tcf7l2* *^loxp/loxp^* (JAX031436), *Ascl1* *^−/+^* (JAX012881), and *EIIa-Cre* mice (JAX003314) obtained from The Jackson Laboratory, Bar Harbor, ME.was described previously (Lakso et al., 1996; Leung et al., 2007; Angus-Hill et al., 2011; Sokolowski et al., 2015).

### 2.2 Immunohistochemistry

Embryonic brains were fixed in 4% formaldehyde in PBS, pH7.4 for 2–3 h at 4°C, immersed in 30% sucrose, cryosectioned at 25 µm by Leica CM3050 Cryostat, and placed on glass slides. Sections were blocked with 2% normal sheep serum (Sigma) in PBS for 1 h, incubated in primary antibody made up in 1% sheep serum, 0.4% Triton X-100 in PBS overnight. The following primary antibodies were used: anti-phospho-Histone H3 (Upstate Biotechnology, 06–570; 1:1000), and anti-BrdU (Sigma, B8434; 1:1000). Following incubation with primary antibody, sections were washed three times with PBS, pH7.4 and incubated with species-specific secondary antibodies conjugated to Alexa Fluor (Molecular Probes, A11034, A11003, A11010, A11029; 1:200). After three washes in PBS, sections were mounted on slides with Fluoromount G (Electron Microscopy Sciences).

### 2.3 BrdU labeling and birthdating

BrdU labeling was performed as described previously (Miller and Nowakowski, 1988), with some modifications. Timed pregnant female mice were injected subcutaneously with 50 mg/kg of 5-bromo-2′-deoxyuridine (Sigma). For pulse labeling experiments, mice were sacrificed after 2–24 h. For birth dating experiments, mice were sacrificed at E18.5. Cryostat sections were permeabilized in 0.4% Triton X-100, treated with 50% formamide/2x SSC for 2 h at 65°C, 2x SSC for 10 min, 2 M HCl for 1 h at 37°C with, washed twice for 10-min in 0.1 M sodium borate, and stained with anti-BrdU antibody.

### 2.4 Cell counting

For quantitation of proliferation and BrdU birth dating, images of stained sections were captured using a Leica DMI6000B digital camera (Leica Microsystems), and the number of cells was scored from captured images. Counting grids were set at 200 μm × 200 μm in the SC, and at 100 μm × 100 μm in the IC. In most cases, 20–30 counting sites were evaluated in one section for the region of interest. All data were obtained from at least three samples of each genotype, and tested for significance using a two sample Student's t-test with unequal variances.

### 2.5 *In situ* hybridization

Whole-mount and section in situ hybridization were performed using digoxigenin-UTP-labeled riboprobes essentially as described previously (Tran et al., 2020). Embryonic brains were collected from timed pregnant females, and fixed in 4% paraformaldehyde at 4°C for overnight. Brain tissues were embedded in Tissue-Tek O.C.T. compound, and 25 μm frozen sections were collected. Sections were rehydrated in PBS, and immersed in pre-hybridization buffer (1% SDS, 5x SSC, 50% formamide) for 1 h at 65°C. For riboprobes, template DNAs for *in vitro* transcription by T7 RNA polymerase were generated by using PCR primers, of which sequences were obtained from the Allen Brain Atlas or designed against unique regions of transcripts to avoid cross-reactivity (Supplementary Table S1). The promoter sequence for T7 RNA polymerase (5′-TGT AAT ACG ACT CAC TAT AGG GC-3′) was placed at the 5′ end of reverse primers. DIG-labeled RNA probe was then added in hybridization buffer (1% SDS, 5x SSC, 50% formamide, 200 mg/mL yeast tRNA, 5% heparin). Incubation overnight at 65°C followed washing for 30 min in 1x SSC/50% formamide at 65°C. Slides were then treated with RNase A (20 mg/mL) in a buffer containing 10 mM Tris pH 7.5, 500 mM NaCl, 1 mM EDTA at 37°C for 30 min, washed in 2x SSC for 20 min at 65°C, and then twice for 20 min each in 0.2x SSC. After incubation overnight with anti-DIG AP antibody (1:2500, Roche), slides were washed in MABT and equilibrated in NTM for 10 min. Color detection was performed using BM purple (Roche). At least three independent experiments were performed for any given riboprobes, which showed highly reproducible gene expression data.

### 2.6 Statistical analysis

**FIGURE 1:**
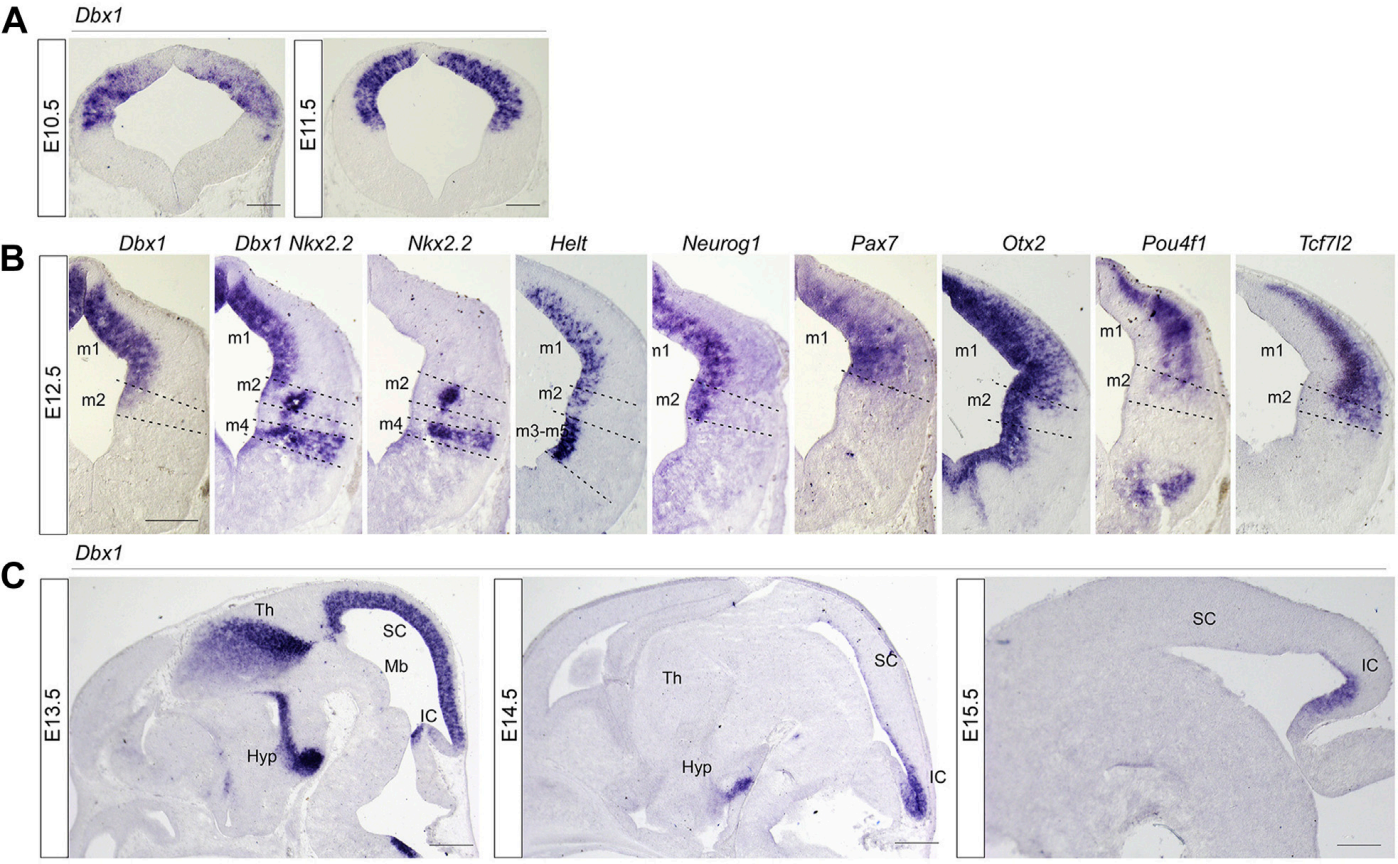
Spatial and temporal pattern of *Dbx1* expression in embryonic mouse midbrain compared with expression of neural subtype markers. **(A)** Representative transverse midbrain sections of wild-type mouse embryos hybridized with *Dbx1* riboprobe at E10.5 and E11.5. **(B)** *In situ* hybridization (ISH) with *Dbx1*, *Nkx2.2*, *Helt*, *Neurog1*, *Pax7*, *Otx2*, *Pou4f1*, and *Tcf7l2* probes on transverse sections of E12.5 wild-type embryos. **(C)** ISH with *Dbx1* probe on sagittal sections of wild-type embryos at E13.5 –E15.5. m1-m5, dorsoventral midbrain domains 1-5. Hyp, hypothalamus; IC inferior colliculus; Mb, midbrain; SC, superior colliculus, Th, thalamus. Scale bars, A = 100 μm, B = 200 μm, C = 400 μm.

**FIGURE 2:**
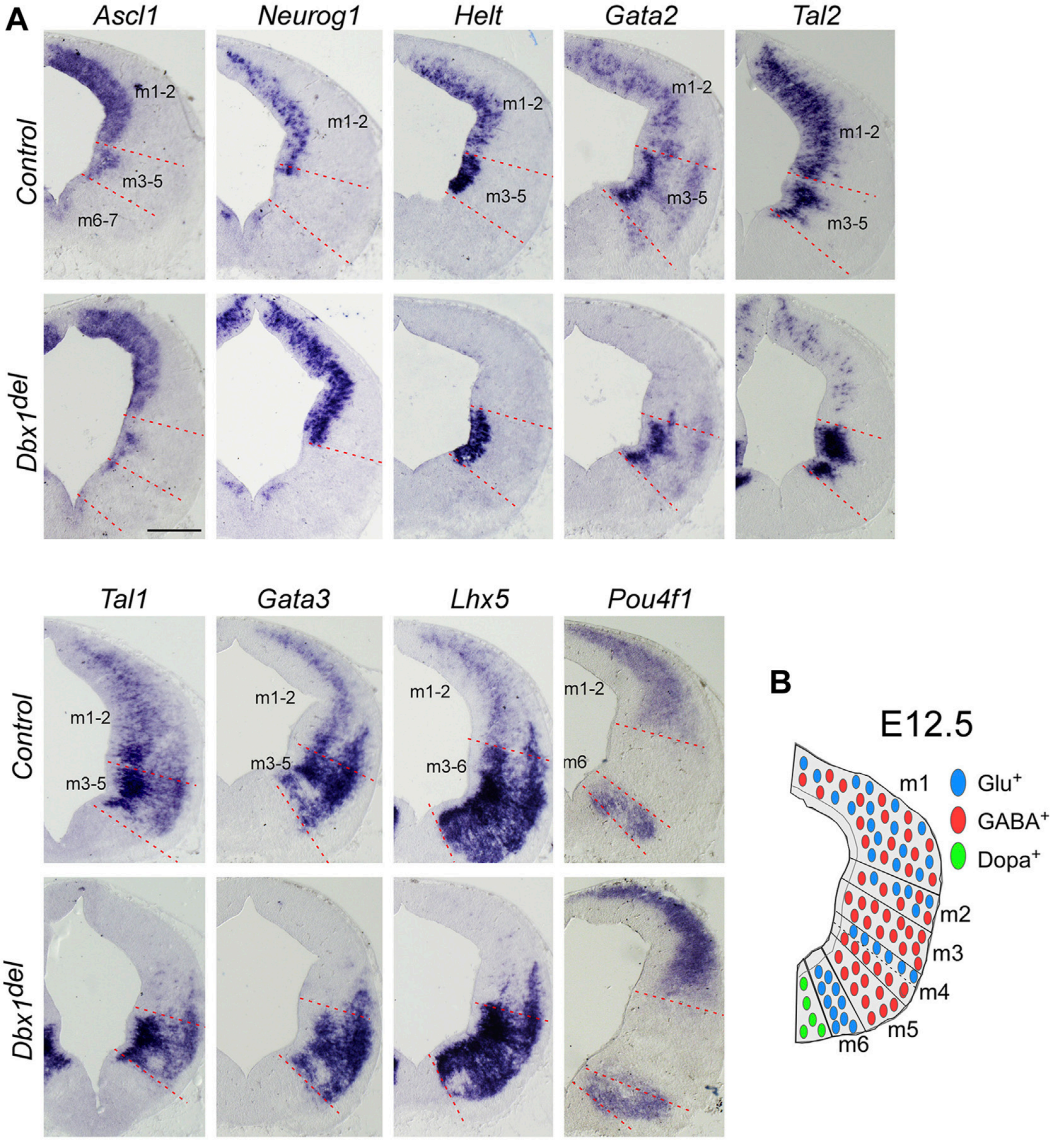
*Dbx1* is required for selection of GABAergic over glutamatergic neuronal fate in the m1 and m2 dorsoventral midbrain domains **(A)** ISH on midbrain transverse sections of control and *Dbx1* ^*del*^ embryos at E12.5. *Ascl1*, *Neurog1*, and *Helt* were expressed in the ventricular progenitor region, while *Gata2* and *Tal2* expression were observed in the ventricular and intermediate zones. *Tal1*, *Gata3*, *Lhx5*, and *Pou4f1* were detectable in the intermediate and postmitotic marginal zones. Loss of *Dbx1* resulted in severe downregulation of GABAergic lineage genes *Helt*, *Gata2*, *Tal2*, *Tal1*, *Gata3*, and *Lhx5*, while glutamatergic lineage genes *Neurog1* and *Pou4f1* were upregulated. **(B)** Schematic view of E12.5 midbrain depicting domains m1– m7 and the distribution of glutamatergic (blue circles), GABAergic (red circles), and dopaminergic (green circles) neurons. Scale bar, A = 200 μm.

**FIGURE 3:**
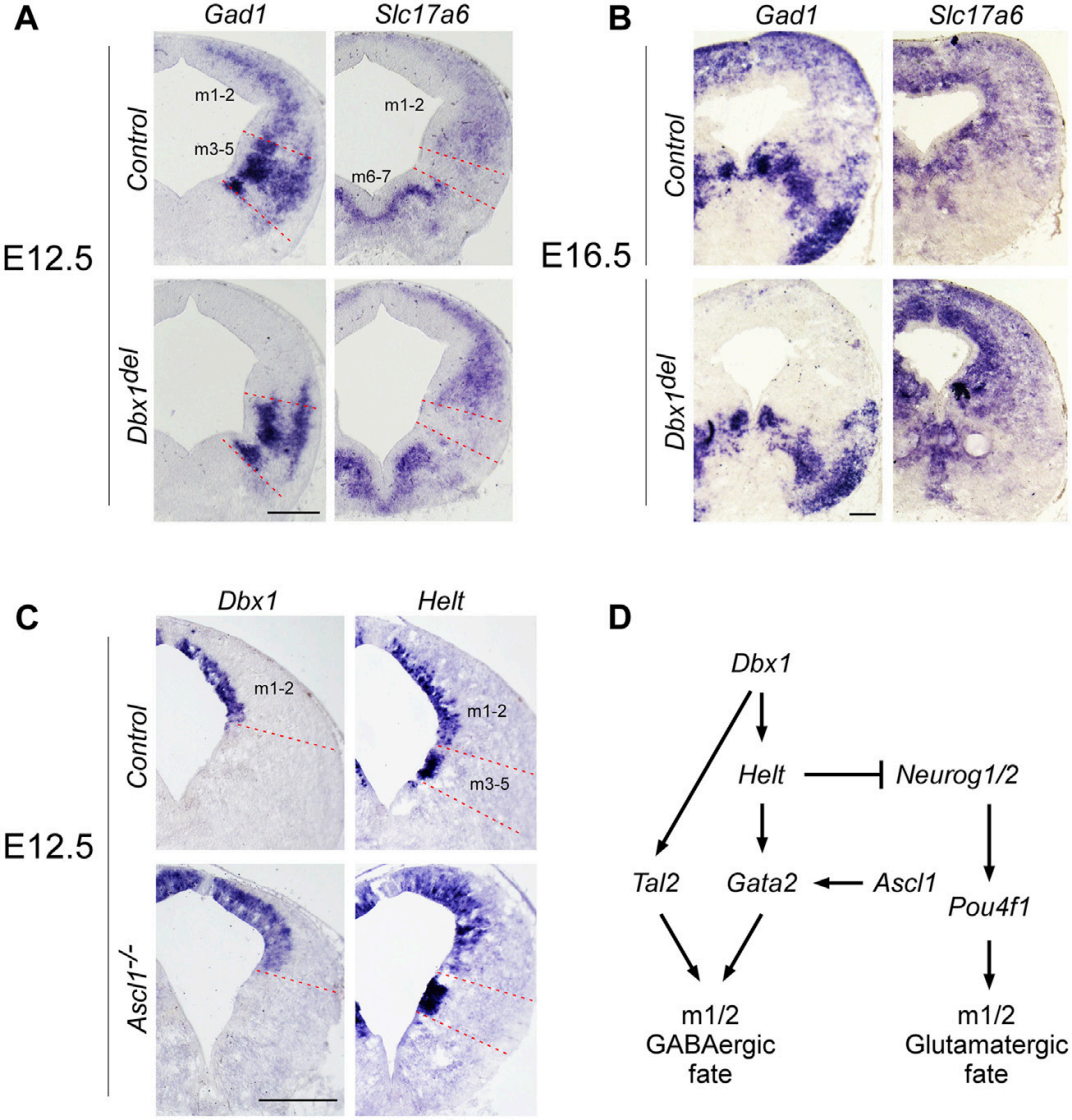
Distinct regulatory pathways control GABAergic neuronal fate determination in the m1 and m2 domains **(A,B)** ISH on midbrain transverse sections of control and *Dbx1* ^*del*^ embryos at E12.5 **(A)** and E16.5 **(B)**. In control embryos, *Gad1* and *Slc17a6* expression were commonly detectable in m1 and m2 domains of the midbrain. Inactivation of *Dbx1* downregulated *Gad1* staining selectively in m1-m2 domains, but not in m3-m5 domains. By contrast, *Slc17a6* expression was upregulated in m1-m2 domains of *Dbx1* ^*del*^ mutants. **(C)** Representative transverse midbrain sections of wild-type and *Ascl1* ^*−/−*^ mouse embryos hybridized with *Dbx1* and *Helt* riboprobes. **(D)** Schematic showing genetic cascades that control GABAergic neuronal specification in the dorsal midbrain. Scale bars, A, C = 200 μm, B = 400 μm.

**FIGURE 4:**
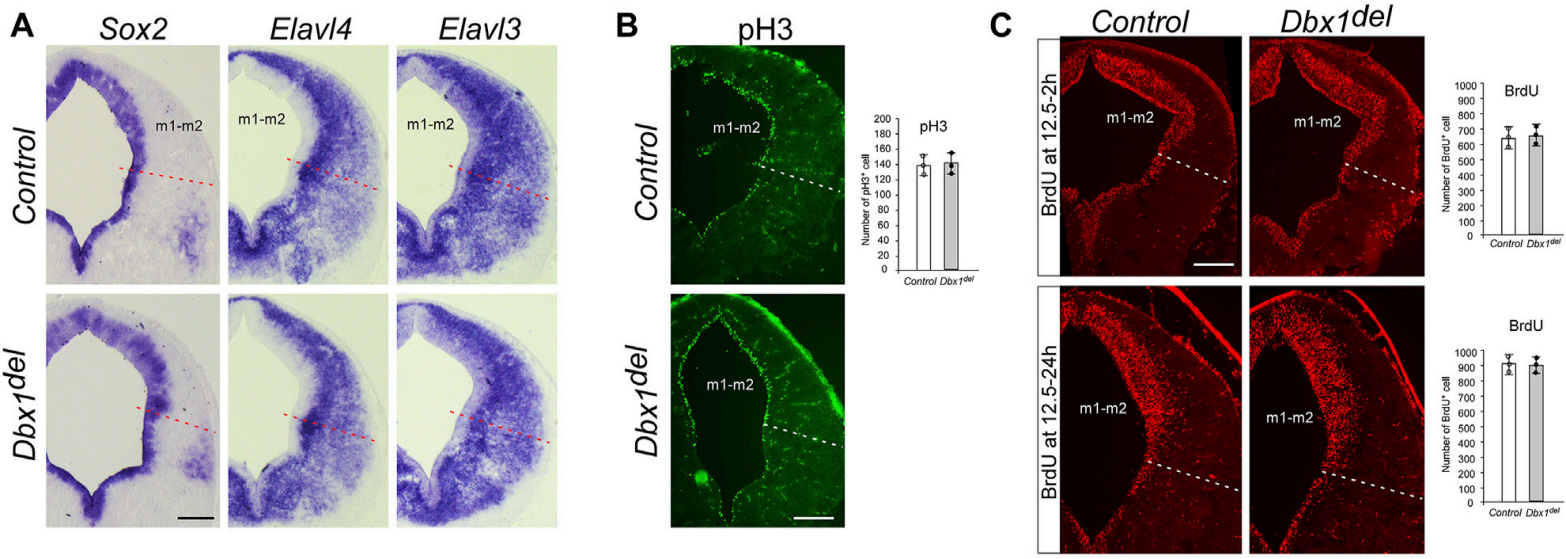
Unaltered progenitor cell proliferation and neurogenesis in *Dbx1* ^*del*^ midbrain **(A)** ISH of *Sox2* and *Elavl3/4* (HuC/D) on transverse sections of E12.5 embryos demonstrates no obvious difference between control and *Dbx1* ^*del*^ midbrains. **(B)** Immunohistochemistry (IHC) staining for phospho-histone H3 (pH3) on transverse sections of E12.5 midbrain. The number of pH3-positive cells counted from the ventricular zone of the m1-m2 domain (three sections from three embryos for each genotype) **(C)** Incorporation of BrdU in E12.5 control and *Dbx1* ^*del*^ midbrain during a 2–24 h labeling pulse detected by anti-BrdU IHC (three sections from three embryos for each genotype). Scale bars, 200 μm.

**FIGURE 5:**
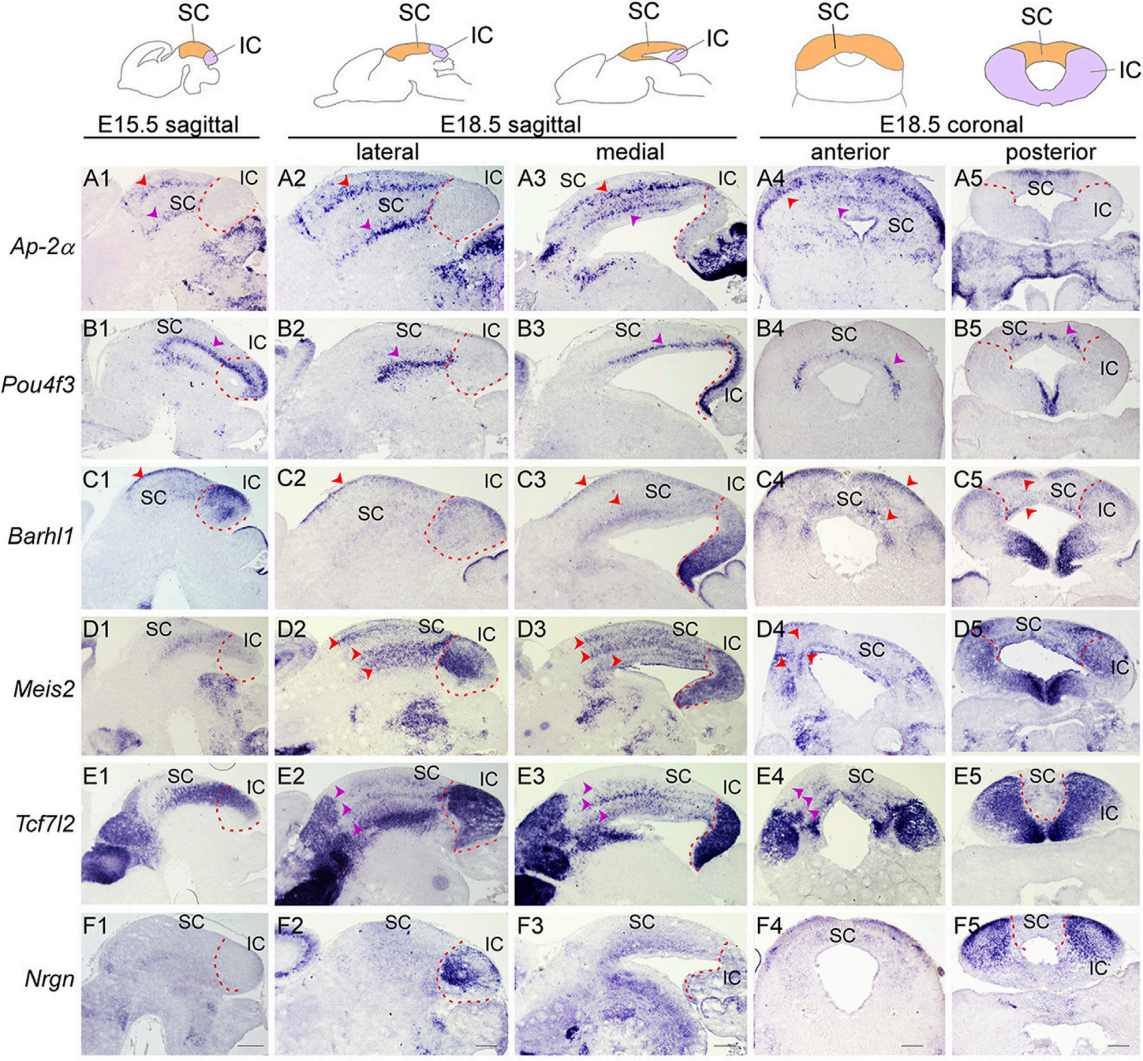
Molecular organization of the developing SC and IC (Top) Schematic view of E15.5 and E18.5 brain sections showing the superior colliculus (SC, orange) and inferior colliculus (IC, purple). **(A–F)** ISH with *Ap-2α* **(A)**, *Pou4f3* **(B)**, *Barhl1* **(C)**, *Meis2* **(D)**, *Tcf7l2* **(E)** and *Nrgn* **(F)** probes on sagittal sections of E15.5 wild-type embryos, and sagittal and transverse sections of E18.5 wild-type embryos. Multiple cellular layers of the SC can be identified by the expression of *Ap-2α*, *Barhl1*, *Pou4f3*, *Meis2*, and *Tcf7l2* (arrowheads). *Meis2* and *Tcf7l2* expression are present in the almost entire region of the IC, while *Barhl1*, *Pou4f3*, and *Nrgn* are confined to discrete regions of the IC. Dashed curves delimit the IC region. Scale bars, 400 μm.

**FIGURE 6:**
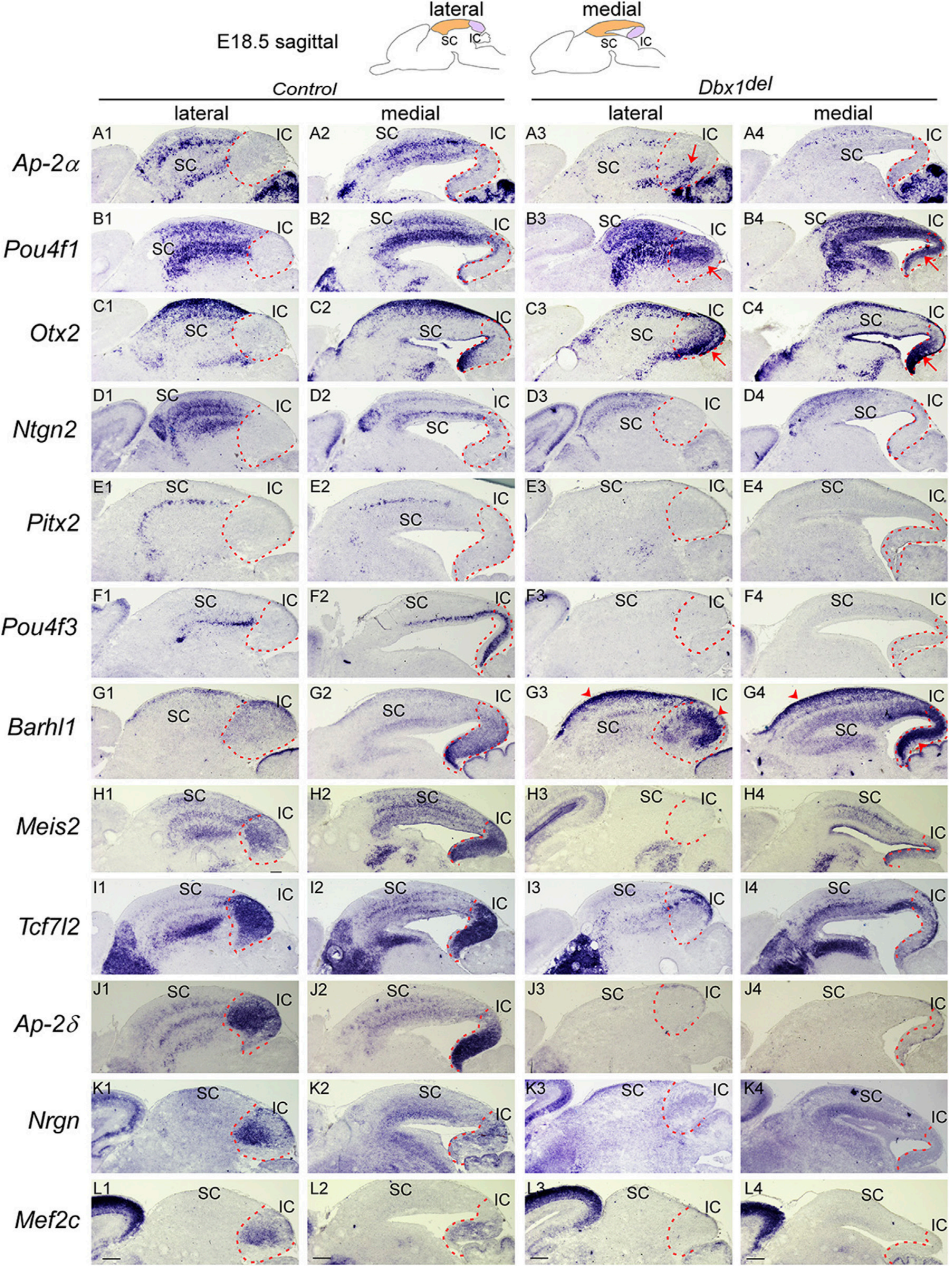
Loss of *Dbx1* remarkably affects identity and differentiation of the superior and inferior colliculi **(A–L)** Section ISH on midbrain sagittal sections of control and *Dbx1* ^*del*^ embryos at E18.5 with *Ap-2α* **(A)**, *Pou4f1* **(B)**, *Otx2* **(C)**, *Ntng2* **(D)**, *Pitx2* **(E)**, *Pou4f3* **(F)**, *Barhl1* **(G)**, *Meis2* **(H)**, *Tcf7l2* **(I)**, *Ap-2δ* **(J)**, *Nrgn* **(K)**, *Mef2c* **(L)**. Red arrows indicate ectopic upregulation of *Otx2*, *Pou4f1*, and *Ap-2α* in the IC. Dashed curves delimit the IC region. Scale bars, 400 μm.

**FIGURE 7:**
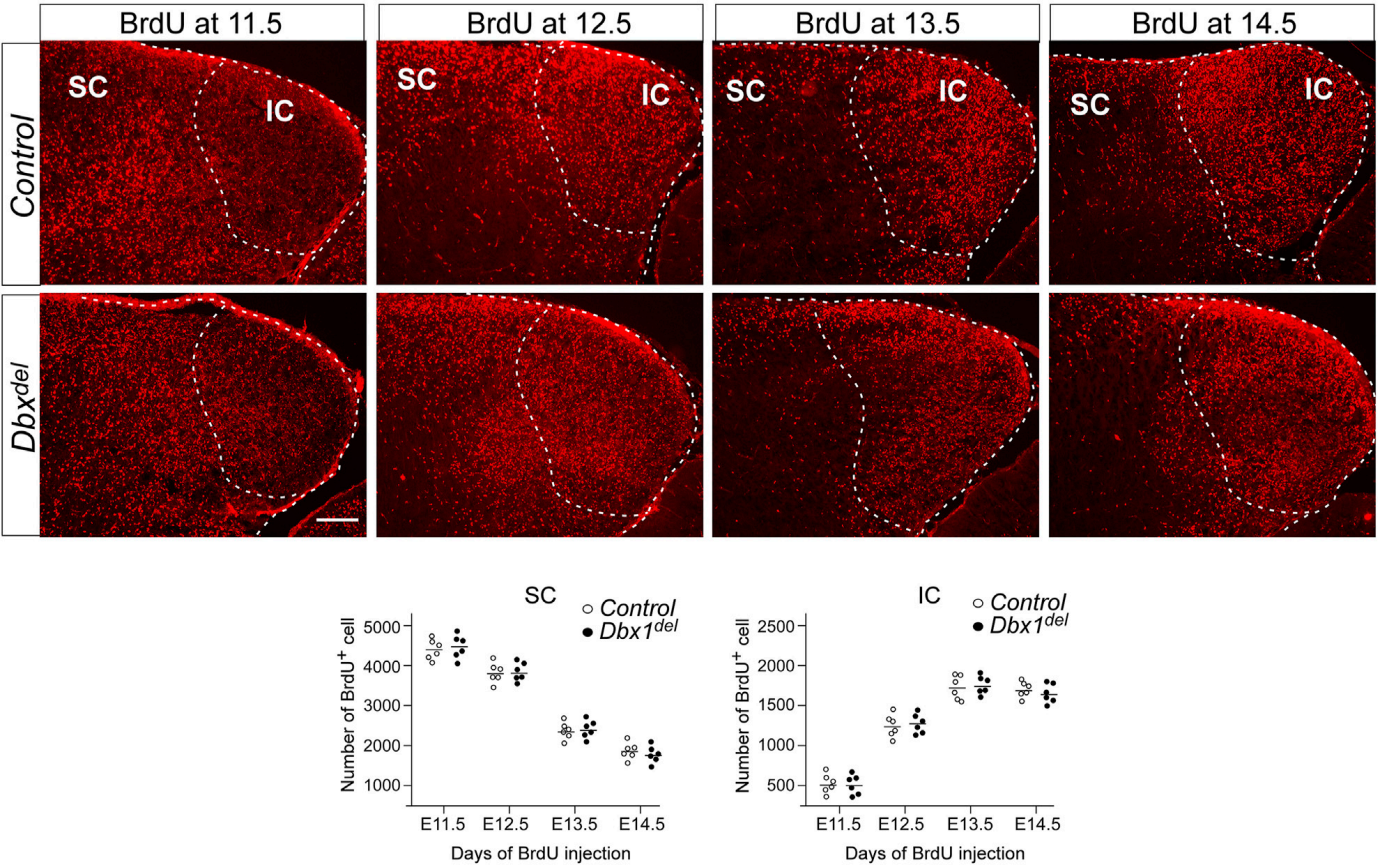
Birthdating of IC and SC neurons Pregnant mice were injected with BrdU at E11.5-E14.5, and brains were harvested at E18.5. For counting of BrdU^+^ cells, 10–20 counting sites (counting grid for SC, 200 μm × 200 μm; for IC, 100 μm × 100 μm) were evaluated in one section. The values were calculated from five sections of six embryos for each genotype. Scale bars, 200 μm.

**FIGURE 8:**
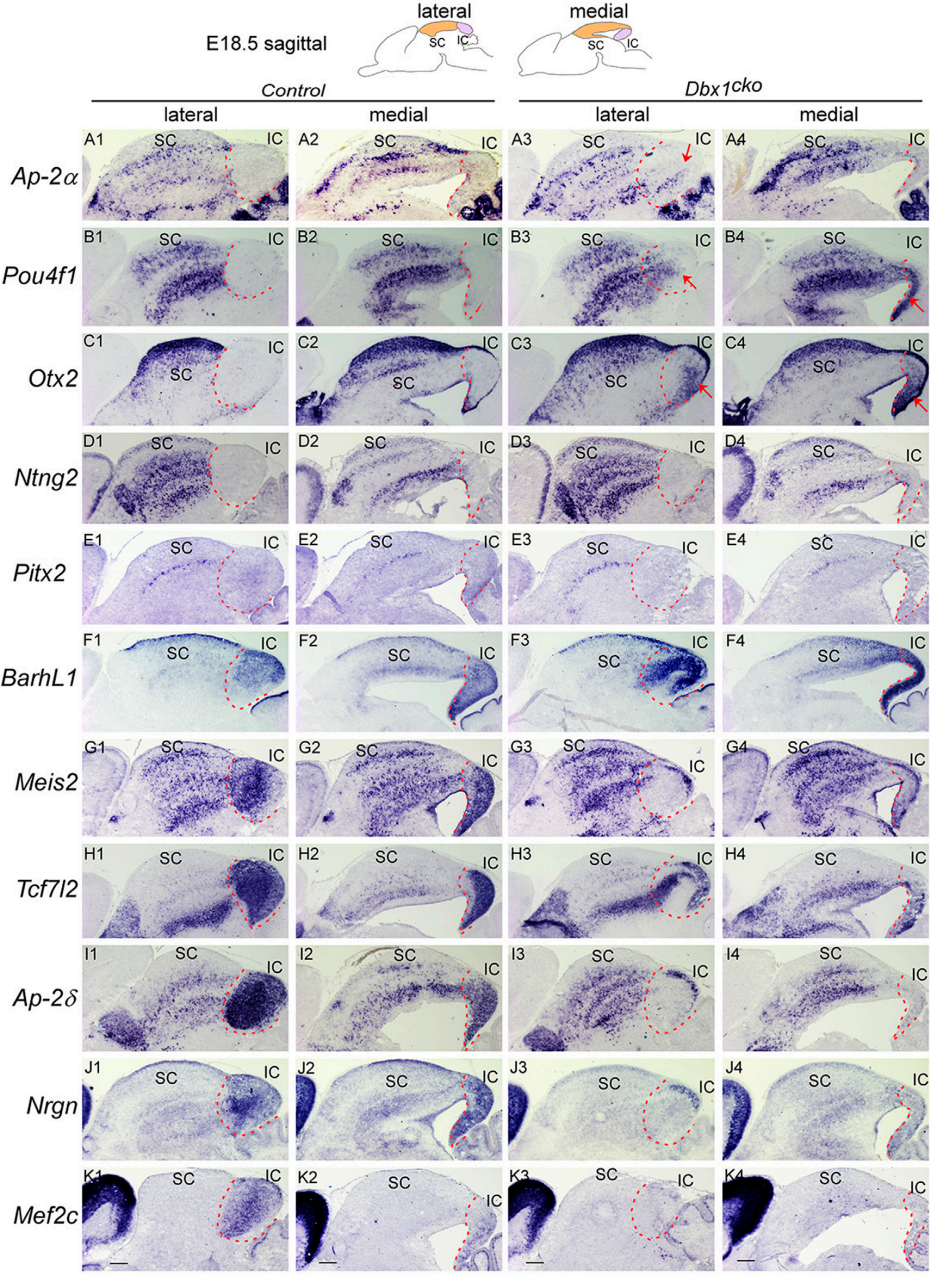
*Dbx1* is required during distinct temporal windows for development of different structures in the dorsal midbrain Section ISH on midbrain sagittal sections of control and *Dbx1* ^*cko*^ embryos at E18.5 with *Ap-2α* **(A)**, *Pou4f1* **(B)**, *Otx2* **(C)**, *Ntng2* **(D)**, *Pitx2* **(E)**, *Barhl1* **(F)**, *Meis2* **(G)**, *Tcf7l2* **(H)**, *Ap-2δ* **(I)**, *Nrgn* **(J)**, *Mef2c* **(K)**. Red arrows indicate ectopic upregulation of *Otx2*, *Pou4f1*, and *Ap-2α* in the IC. Dashed curves delimit the IC region. Scale bars, 400 μm.

**FIGURE 9:**
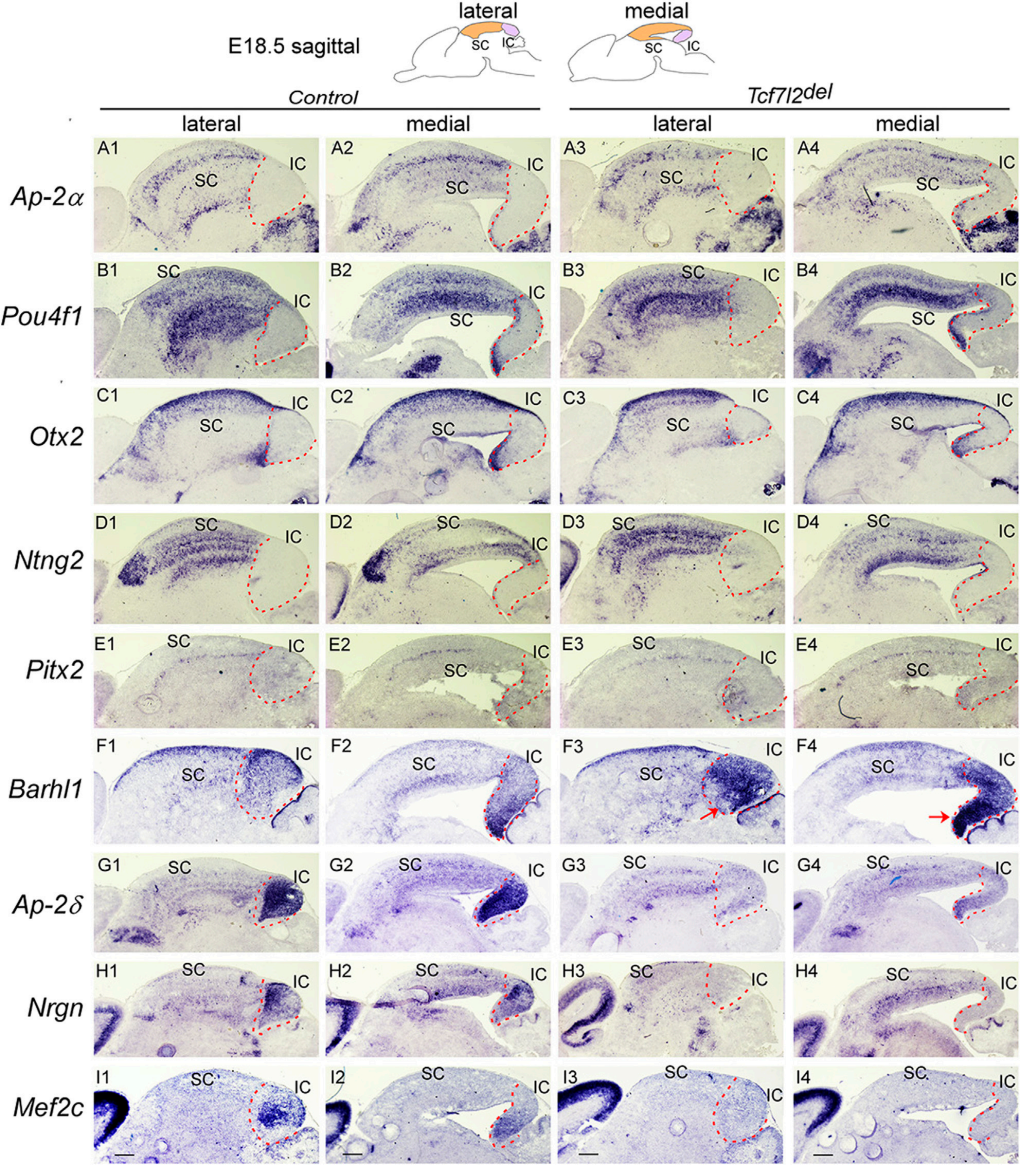
IC differentiation is impaired in the absence of *Tcf7l2* Section ISH on midbrain sagittal sections of control and *Tcf7l2* ^*del*^ embryos at E18.5 with *Ap-2α* **(A)**, *Pou4f1* **(B)**, *Otx2* **(C)**, *Ntng2* **(D)**, *Pitx2* **(E)**, *Barhl1* **(F)**, *Ap-2δ* **(G)**, *Nrgn* **(H)**, *Mef2c* **(I)**. Red arrows (F3,F4) indicate ectopic upregulation of *Barhl1* in the IC. Dashed curves delimit the IC region. Scale bars, 400 μm.

All mutant phenotypes reported in *Ascl1* ^*-/-*^, *Dbx1* ^*del*^, *Dbx1* ^*cko*^, and *Tcf7l2* ^*del*^ embryos were completely penetrant. The number of independent values in each experiment were as follows: Figure 1: n = 3 for E10.5, E11,5 and E12.5 for ISH coronal and sagittal sections. Figure 2: n = 3 for E12.5 ISH. Figure 3: n = 3 for E12.5 and E16.5 coronal ISH. Figure 4: n = 3 for E12.5 ISH and IHC with PH3. Three independent biological replicates for BrdU labelling at E12.5 with 2 and 24 h of incubation. Three sections of three embryos from each genotype were selected to count pH3^+^ and BrdU^+^. Figure 5: n = 3 for E15.5 and E18.5 ISH. Figure 6: n = 3 for E18.5 ISH. Figure 7: three independent biological replicates for BrdU birth-dating tracing from E11.5, E12.5, E13.5 and E14.5. For counting BrdU^+^ cells, 10–20 counting site (counting grid for SC: 200 μm × 200 μm; for IC: 100 μm × 100 μm) were evaluated in one section. The value was calculated from five sections of six embryos for each genotype. Figure 8: n = 3 for E18.5 ISH. Figure 9: n = 3 for E18.5 ISH.

## 3 Results

### 3.1 *Dbx1* is an m1/2 dorsoventral domains-specific determinant of the GABAergic neuron fate

In order to understand the role of *Dbx1* in mesencephalic neuronal differentiation, we first compared the distribution pattern of *Dbx1* with those of transcription factors potentially involved in the specification of mesencephalic progenitors and derived neurons. *Dbx1* expression was detectable in the dorsolateral wall of the mesencephalic neuroepithelium at E9.5 ∼ E10.5 (Lu et al., 1992; Shoji et al., 1996) (Figure 1A), and subsequently, at E11.5, was limited to the ventricular zone (Figure 1A). At E12.5-E13.5, *Dbx1* was co-expressed in the m1-m2 dorsoventral progenitor domains of the dorsal midbrain with *Ascl1*, *Helt*, *Neurog1*, *Pax7*, and *Otx2* (Figure 1B; Figures 2A, B). At E14.5-E15.5, there was a marked reduction in *Dbx1* expression in the SC, but its expression persisted in the IC (Figure 1C).

The results of our gene expression experiments demonstrated that *Dbx1* is actively expressed in the dorsal midbrain progenitors at the time when their dorsoventral identity is being established. Thus we intended to determine whether *Dbx1* is involved in early regional patterning and neurogenesis in the dorsal midbrain. To achieve these goals, we analyzed *Dbx1* ^*del*^ mutants in which *Dbx1* is inactivated at the pre-implantation stage (Tran et al., 2023). Recent studies demonstrated that GABAergic and glutamatergic neurons derived from the progenitor domains of the developing midbrain are regulated by combinations of multiple transcription factors acting as fate selectors. GABAergic fate determination requires the function of *Ascl1* and *Helt* in the progenitor region and then subsequent activation of the post-mitotic selectors *Gata2* and *Tal2* (Miyoshi et al., 2004; Guimera et al., 2006; Nakatani et al., 2007; Kala et al., 2009; Peltopuro et al., 2010; Achim et al., 2013; Wende et al., 2015), whereas the glutamatergic lineage fate is initiated by progenitors expressing *Neurog1*, which is required for post-mitotic activation of *Pou4f1* (*Brn3a*) (Guimera et al., 2006; Nakatani et al., 2007). In *Dbx1*-deficient embryos, *Ascl1* expression was apparently normal, whereas the expression of *Helt*, *Gata2*, and *Tal2* was lost specifically in the m1-m2 dorsoventral domains despite normal expression in the ventral domains (Figure 2A). Consistently, the post-mitotic GABAergic neuronal markers *Gata3*, *Tal1*, and *Lhx5* were not detectable in the m1-m2 domain of *Dbx1* ^*del*^ mutants (Figure 2A). By contrast, loss of *Dbx1* resulted in upregulation of the glutamatergic lineage markers, *Neurog1* and *Pou4f1* in the m1-m2 domain despite normal expression in the ventral region. These defects in GABAergic differentiation were similarly detected also in the posterior-most region of the midbrain (Supplementary Figure S1). Next, we examined the neurotransmitter profile in *Dbx1*-deficient embryos. GABAergic neurons express genes encoding the glutamate decarboxylase enzyme, *Gad1/2*, while glutamatergic neurons express the vesicular glutamate transporter *Slc17a6*. Compared to controls, in *Dbx1* ^*del*^ mutants, there was an almost complete loss of *Gad1* staining in the m1-m2 domain, while *Slc17a6* expression was increased (Figures 3A, B; Supplementary Figure S2).

To determine a genetic hierarchy, we analyzed *Dbx1* and *Helt* expression in *Ascl1* mutants. Loss of *Ascl1* did not alter *Dbx1* or *Helt* expression, although the progenitor domain expanded due to a failure in cell cycle exit (Peltopuro et al., 2010; Wende et al., 2015) (Figure 3C), implicating two parallel (*Dbx1* vs. *Ascl1*) regulatory strategies for GABAergic fate determination in the dorsal midbrain (Figure 3D). Notably, as *Tal2* is regulated by *Dbx1* (Figure 2A), but not by *Ascl1*, *Helt* or *Gata2* in the dorsal midbrain (Kala et al., 2009; Peltopuro et al., 2010; Wende et al., 2015), these observations demonstrate that *Dbx1* regulates GABAergic fate via two different downstream cascades, *Helt*-*Gata2* vs. *Tal2* (Figure 3D).

### 3.2 *Dbx1* is not involved in progenitor cell proliferation and neurogenesis

Since *Dbx1* is selectively expressed in the ventricular progenitor region and the onset of its transcription precedes neurogenesis, we examined the general properties of m1-m2 progenitor cells in *Dbx1* mutant embryos. During neural development, Sox2 acts to maintain an undifferentiated cell state, and its expression becomes restricted to early progenitor and neural stem cells (Bylund et al., 2003; Graham et al., 2003; Cavallaro et al., 2008; Hagey and Muhr, 2014). The neuron-specific RNA binding proteins HuC/D encoded by *Elavl3/4* genes are restricted to post-mitotic neurons (Szabo et al., 1991; Okano and Darnell, 1997). In *Dbx1* mutant embryos, we detected no major difference in the thickness of the *Sox2* ^+^ ventricular zone or the *Elavl3/4* ^+^ (*HuC/D*) marginal zone (Figure 4A). Consistently, there was no major changes in the numbers of phosphor-histone H3^+^ mitotic cells or BrdU^+^ S-phase cells (Figures 4B, C). Thus, these results suggest that loss of *Dbx1* does not impair generic neurogenic properties.

### 3.3 *Dbx1* is required for identity and differentiation of the SC and IC and prevents IC into SC fate switch

Early failure in GABAergic fate determination in the dorsal midbrain of *Dbx1* mutants led us to ask whether *Dbx1* is also required for morphological maturation of the dorsal midbrain. We first characterized molecular features of the dorsal midbrain at E15.5, when the IC and SC, two morphologically distinct compartments of the dorsal midbrain, are detectable, and the SC forms multiple cellular and fibrous layers. *Ap-2α* expression was restricted to the SC and not detectable in the IC (Figure 5A1). *Pou4f3*, *Barhl1*, *Meis2*, and *Tcf7l2* expression were continuous between the SC and IC at E15.5 (Figures 5B1–E1). At E18.5, the overall patterns of *Ap-2α* and *Barhl1* expression were maintained (Figures 5A2, A3, C2, C3), but the lateral region of the IC was completely devoid of *Pou4f3* expression (Figures 5B2). *Meis2* and *Tcf7l2* displayed discontinuous and discrete expression pattern between the SC and IC (Figures 5D2, D3, E2, E3), and *Nrgn* expression was strongly detected in the lateral region of the IC (Figures 5F2, F5). The SC develops a layered structure with a number of layers that varies by species. The SC layers can be grouped into the superficial layers (zonal, superficial gray, and optic layers) and the deeper layers (intermediate gray, intermediate white, deep gray, and deep white layers). *Ap-2α*, *Barhl1*, *Meis2*, and *Tcf7l2* expression were detectable in the superficial and deeper layers (Figures 5A2–A4, C2–C4, D2–D4, E2–E4), but *Pou4f3* expression was specific for the deep gray layer (Figures 5B2–B4). Besides, molecularly distinct regions of the IC can be identified by differential gene expression of *Pou4f3*, *Barhl1*, *Meis2*, *Tcf7l2* and *Nrgn* (Figures 5B5–F5). The spatial distribution patterns of these genes observed at E18.5 were maintained until postnatal or adult stages (Allen Brain Atlas, https://mouse.brain-map.org/), indicating that the molecular organization of the SC and IC are established during a narrow window of embryonic development (E15.5–E18.5).

To investigate whether *Dbx1* regulates differentiation of the SC and IC, we analyzed *Dbx1* ^*del*^ mutants at E18.5, when morphological maturation is established. In the SC of *Dbx1* ^*del*^ embryos, *Ap-2α* expression was severely downregulated despite strong staining in the cerebellum (Figures 6A3, A4). *Pou4f1* (*Brn3a*) was expressed in multiple layers of control SC (Figures 6B1, B2), but its striped pattern was disrupted in the SC of *Dbx1* ^*del*^ embryos (Figures 6B3, B4). *Otx2* expression, which was confined to the superficial layers of control SC, was downregulated in the absence of *Dbx1* (Figures 6C3, C4). In control embryos, *Ntng2* expression was detected in both superficial and deep layers of the SC (Figures 6D1, D2), while in the SC of *Dbx1* ^*del*^ embryos, the striped pattern of *Ntng2* expression was abolished (Figures 6D3, D4). *Pitx2* and *Pou4f3*, which normally mark the intermediate grey and deep grey layers, respectively (Figures 6E1, E2, F1, F2), were not detected in the SC of *Dbx1* ^*del*^ embryos (Figures 6E3, E4, F3, F4). Notably, *Pitx2* is identified as a target of *Gata2* and required for GABAergic neuron differentiation in the SC (Kala et al., 2009; Waite et al., 2011). *Barhl1*, which marks the zonal layer, was strongly upregulated in *Dbx1* ^*del*^ mutants (Figures 6G3, G4, arrowheads). Targeted deletion of *Barhl1* have been shown to disrupt the formation of the SC layers (Martin et al., 2004; Li and Xiang, 2006; Masullo et al., 2019). *Meis2*, *Tcf7l2*, and *Ap-2δ*, which are normally expressed in multiple layers of the SC, were almost absent or downregulated in *Dbx1* ^*del*^ mutants (Figures 6H3, H4, I3, I4, J3, J4). A previous report demonstrated that loss of *Ap-2δ* results in a diminished IC due to abnormal apoptotic cell death (Hesse et al., 2011). Together, these observations indicate that *Dbx1* is critical for normal SC laminar organization.

In the IC of *Dbx1* ^*del*^ embryos, expression of *Pou4f3*, *Meis2*, *Tcf7l2*, *Ap-2δ*, *Nrgn*, and *Mef2c* genes were completely absent or severely downregulated (Figures 6F3, F4, H3, H4, I3, I4, J3, J4, K3, K4, L3, L4). By contrast, *Barhl1* expression was strongly upregulated in the IC of *Dbx1* ^*del*^ embryos (Figures 6G3, G4, arrowheads). Surprisingly, loss of *Dbx1* resulted in strong ectopic activation of SC-specific genes such as *Ap-2α*, *Otx2* and *Pou4f1* in the IC (Figures 6A3, A4, B3, B4, C3, C4, arrows), indicating that *Dbx1* inactivation de-represses a SC-like fate in the IC. Therefore, it is likely that one of the crucial functions of *Dbx1* is to prevent IC from acquiring SC identity.

We next examined whether abnormalities observed in the SC and IC might be associated with a defect in neuronal migration. To estimate the relationships between birthdates and destinations of the dorsal midbrain neurons, we labeled newly born cells with BrdU at E11.5 ∼ E14.5, when neurogenesis in the dorsal midbrain occurs (Cowan et al., 1968; Edwards et al., 1986; Tan et al., 2002). Harvesting embryos at E18.5, we counted labeled cells, having divided the SC into 20 equal zones and the IC into 10 equal zones. In control embryos, more of the earliest generated cells were situated in the SC than in the IC, while the majority of the latest born cells were situated in the IC (Figure 7). These observations were in agreement with previous findings that SC neurons are born earlier than IC neurons (Cowan et al., 1968; Altman and Bayer, 1981b; 1981a; Edwards et al., 1986). We did not observe significant differences in the distribution of BrdU^+^ cells in the SC and IC between controls and *Dbx1* ^*del*^ mutants (Figure 7), suggesting that loss of *Dbx1* does not affect cell migration in the developing dorsal midbrain.

### 3.4 Distinct temporal requirements for *Dbx1* in the regulation of IC and SC differentiation

Given that *Dbx1* expression persists at high levels in the dorsal midbrain during late gestation, our observations raise an intriguing possibility that *Dbx1* has a continued role in IC and SC neuron development during embryogenesis. To determine whether the dependency of dorsal midbrain development on *Dbx1* is regulated temporally, we analyzed *Dbx1* ^*cko*^ mutants in which *Dbx1* function in the midbrain is inactivated at E12.5 (Tran et al., 2023). In contrast to *Dbx1* ^*del*^ embryos, *Dbx1* ^*cko*^ mutants displayed no major alteration in expression of SC-specific genes such as *Ap-2α, Pou4f1*, *Otx2*, *Ntng2* and *Pitx2* (Figures 8A3, A4, B3, B4, C3, C4, D3, D3, E3, E4). Compared to controls, we observed no significant change in *Barhl1* expression in the SC of *Dbx1* ^*cko*^ mutants, whereas its expression in the IC was strongly upregulated in a pattern similar to *Dbx1* ^*del*^ embryos (Figures 8F1–F4). In additions, expression of *Meis2*, *Tcf7l2*, and *Ap-2δ* genes were significantly lost in the IC of *Dbx1* ^*cko*^ mutants despite similar expression in the SC (Figures 8G1–G4, H1–H4, I1–I4). Like in *Dbx1* ^*del*^ IC, the IC-specific markers *Nrgn* and *Mef2c* expression were almost absent in the *Dbx1* ^*cko*^ IC (Figures 8J3, J4, K3, K4). Moreover, we observed ectopic activation of *Ap-2α, Pou4f1*, and *Otx2* in the IC of *Dbx1* ^*cko*^ mutants in a pattern similar to those in *Dbx1* ^*del*^ embryos (Figures 8A3, B3, B4, C3, C4), indicating that persistent expression of *Dbx1* is required for preventing IC into SC fate switch. Therefore, conditional loss of *Dbx1* at later stages did not affect SC differentiation, but caused IC phenotypes with striking similarity to those of *Dbx1* ^*del*^ mutants. These data suggest that SC differentiation is dependent on the early function of *Dbx1*, and the IC requires early and prolonged action for its normal specification and morphological maturation.

We also examined early regional patterning and neuronal specification in the dorsal midbrain of *Dbx1* ^*cko*^ mutant embryos. Compared to controls, we observed no substantial changes in the expression of *Helt*, *Gata2*, *Tal2*, *Tal1*, *Gad1*, and *Slc17a6* in the developing midbrain of *Dbx1* ^*cko*^ mutants (Supplementary Figure S3), indicating that specification of GABAergic neuronal identity is dependent on early *Dbx1* activity.

### 3.5 *Tcf7l2* is required for differentiation of the IC, but not the SC

Previously, we showed that *Tcf7l2* mediates the function of *Dbx1* in the regulation of IC survival (Tran et al., 2023). To address whether *Tcf7l2* is involved in embryonic midbrain patterning and IC/SC differentiation, we first examined the temporal and spatial pattern of *Tcf7l2* expression in the developing midbrain. *Tcf7l2* transcription in the midbrain was detectable after E10.5, and limited to m1-m2 post-mitotic regions (Supplementary Figure S4). *Tcf7l2* expression then persisted in the SC and IC during later embryonic and adult stages (Supplementary Figure S4). To address whether *Tcf7l2* is involved in regulating GABAergc neuronal fate in the dorsal midbrain, we analyzed *Tcf7l2* ^*del*^ mutants in which *Tcf7l2* is inactivated at the pre-implantation stage (Tran et al., 2023). In *Tcf7l2* ^*del*^ mutants, there was no significant alteration in the expression of GABAergic lineage markers such as *Ascl1, Helt*, *Gata2/3*, *Tal1/2*, and *Gad1* (Supplementary Figure S5), indicating that *Tcf7l2* is not required for specifying GABAergic neuronal identity. To determine whether *Tcf7l2* regulates maturation of the dorsal midbrain into SC and IC, we next examined the expression of molecular markers for the IC and SC. We observed no major difference in expression of *Ap-2α*, *Pou4f1*, *Otx2*, *Ntng2*, and *Pitx2* in the SC of *Tcf7l2* ^*del*^ mutants compared with that in controls (Figures 9A1–A4, B1–B4, C1–C4, D1–D4, E1–E4). *Barhl1* expression was similarly detected in the SC of control and *Tcf7l2* ^*del*^ mutants, while its expression was strongly upregulated in the IC of *Tcf7l2* ^*del*^ mutants (Figures 9F1–F4). Furthermore, expression of IC markers such as *Ap-2δ*, *Nrgn*, and *Mef2c* were severely downregulated in *Tcf7l2* ^*del*^ mutants (Figures 9G1–G4, H1–H4, I1–I4), indicating that loss of *Tcf7l2* leads to a phenotype reminiscent of *Dbx1* ^*cko*^ mutants. These data suggest that *Tcf7l2* is selectively required for differentiation of the IC, but not the SC. However, unlike *Dbx1* mutants, there was no ectopic activation of SC-specific markers in the IC of in *Tcf7l2* ^*del*^ mutants (Figures 9A3, A4, B3, B4, C3, C4). Therefore, the function of *Dbx1* to promote IC differentiation is not likely to be mediated entirely by *Tcf7l2* gene.

## 4 Discussion

The present study identifies *Dbx1* as a master regulator of GABAergic neuron fate in the dorsal midbrain. Moreover, our results show that in the m1-m2 domains, *Dbx1* functions upstream of *Helt*, *Gata2*, and *Tal2*, and that *Dbx1* and *Ascl1* act in parallel. Inactivation of *Helt* or *Ascl1* resulted in complete loss of GABAergic neurons in m1-m2 domains, whereas GABAergic neurons in m3-m5 domains were still generated (Guimera et al., 2006; Nakatani et al., 2007; Peltopuro et al., 2010). However, combined deletion of *Helt* and *Ascl1* abrogates induction of GABAergic neurons in all of the m1-m5 domains. Therefore, these observations suggest that GABAergic progenitor specification employs distinct molecular mechanisms depending on the location of the cells’ birth (Wende et al., 2015). Interestingly, loss of *Ascl1* resulted in delayed cell cycle exit and onset of differentiation (Peltopuro et al., 2010), whereas deletion of *Dbx1* or *Helt* did not affect that process (Guimera et al., 2006; Nakatani et al., 2007) (Figure 4), suggesting that neurogenesis and subtype specification including neurotransmitter selection may be separate processes. After cell cycle exit, *Gata2* and *Tal2* function as post-mitotic selectors between GABAergic and glutamatergic neurons. *Gata2* expression was dependent on *Helt* and *Ascl1*, whereas *Tal2* activation required none of those factors except for in the m5 domain (Kala et al., 2009; Peltopuro et al., 2010; Wende et al., 2015). Notably, loss of *Dbx1* abrogated *Tal2* expression in the m1-m2 domains, indicating that *Dbx1* functions to promote GABAergic fate determination at least by controlling two different regulatory axes, the *Helt*/*Gata2*-dependent and *Tal2*-dependent cascades (Figure 3D).

By studying the sequential genetic changes underlying the role of *Dbx1*, we demonstrated that *Dbx1* functions for distinct lengths of time in the development of different dorsal structures of the midbrain. A question arises why the SC require the early activity of *Dbx1*, and IC development is dependent on persistent expression of *Dbx1*. Previous studies showed that growth and expansion of the dorsal midbrain occur in an anterior-to-posterior direction so that the posterior region of the dorsal midbrain continues to divide even after the anterior region ceases proliferation and undergoes neurogenesis (Cowan et al., 1968; Altman and Bayer, 1981b; 1981a; Edwards et al., 1986). Consistently, our BrdU fate mapping experiments also demonstrated that SC neurons are born earlier with peak generation around E11.5 ∼ E12.5, whereas IC neuron generation peaks around E12.5 ∼ E14.5. In agreement with these results, *Dbx1* expression persisted in the IC even after it was excluded from the SC (Figure 1C). These observations together may explain why IC, but not SC requires the prolonged activity of *Dbx1* for normal differentiation.

*Dbx1* plays important roles in the generation of specific subclasses of neurons in a region-specific manner by regulating the expression of effector genes, which work together to control the formation of V0 interneurons in the spinal cord, Pre-Bötzinger complex neurons in the hindbrain, orexigenic neurons in the hypothalamus (Pierani et al., 2001; Bouvier et al., 2010; Gray et al., 2010; Sokolowski et al., 2015)). In this study, we demonstrate that *Tcf7l2* functions as an effector gene to mediate the activity of *Dbx1* for IC differentiation. During embryogenesis, *Dbx1* and *Tcf7l2* expression are restricted to m1-m2 domains, which differentiate into the SC and IC. Loss of *Dbx1* resulted in downregulation of *Tcf7l2* in the SC and IC. However, unlike *Dbx1*, loss of *Tcf7l2* did not affect SC differentiation. A question arises why the IC requires *Tcf7l2* function for its development, and SC development is not dependent on *Tcf7l2*. Wnt/β-catenin signaling controls multiple processes during early midbrain development and dopaminergic (mDA) neuron differentiation. All Wnts that use canonical signaling converge on β-catenin, which activates target genes via Tcf/Lef factors (Liu et al., 2022). Vertebrates encode four *Tcf/Lef* genes (*Tcf7*, *Lef1*, *Tcf7l1*, *Tcf7l2*), which play important roles in mediating the diverse functions of Wnt/β-catenin signaling in different tissues at different stages. The overlapping expression patterns and functions of Tcf/Lef factors preclude an understanding of the specificity of individual factor involvement in neuron development *in vivo*. All four *Tcf/Lef* factors are expressed in the developing midbrain at early stages. Interestingly, later, *Tcf7l2* is the only factor expressed in the IC (Allen Brain Atlas). Therefore, it is likely that functional redundancy may exist during SC development (Cadigan and Waterman, 2012).

## Data Availability

The raw data supporting the conclusion of this article will be made available by the authors, without undue reservation.

## References

[B1] Achim K. Peltopuro P. Lahti L. Li J. Salminen M. Partanen J. (2012). Distinct developmental origins and regulatory mechanisms for GABAergic neurons associated with dopaminergic nuclei in the ventral mesodiencephalic region. Development 139, 2360–2370. 10.1242/dev.076380 22627282

[B2] Achim K. Peltopuro P. Lahti L. Tsai H.-H. Zachariah A. Åstrand M. (2013). The role of Tal2 and Tal1 in the differentiation of midbrain GABAergic neuron precursors. Biol. Open 2, 990–997. 10.1242/bio.20135041 24167708 PMC3798194

[B3] Agoston Z. Schulte D. (2009). Meis2 competes with the Groucho co-repressor Tle4 for binding to Otx2 and specifies tectal fate without induction of a secondary midbrain-hindbrain boundary organizer. Development 136, 3311–3322. 10.1242/dev.037770 19736326

[B4] Altman J. Bayer S. A. (1981a). Time of origin of neurons of the rat inferior colliculus and the relations between cytogenesis and tonotopic order in the auditory pathway. Exp. Brain Res. 42, 411–423. 10.1007/BF00237506 7238680

[B5] Altman J. Bayer S. A. (1981b). Time of origin of neurons of the rat superior colliculus in relation to other components of the visual and visuomotor pathways. Exp. Brain Res. 42, 424–434. 10.1007/BF00237507 7238681

[B6] Angus-Hill M. L. Elbert K. M. Hidalgo J. Capecchi M. R. (2011). T-cell factor 4 functions as a tumor suppressor whose disruption modulates colon cell proliferation and tumorigenesis. Proc. Natl. Acad. Sci. 108, 4914–4919. 10.1073/pnas.1102300108 21383188 PMC3064334

[B7] Basson M. A. Echevarria D. Petersen Ahn C. Sudarov A. Joyner A. L. Mason I. J. (2008). Specific regions within the embryonic midbrain and cerebellum require different levels of FGF signaling during development. Development 135, 889–898. 10.1242/dev.011569 18216176 PMC2555978

[B8] Bouvier J. Thoby-Brisson M. Renier N. Dubreuil V. Ericson J. Champagnat J. (2010). Hindbrain interneurons and axon guidance signaling critical for breathing. Nat. Neurosci. 13, 1066–1074. 10.1038/nn.2622 20680010

[B9] Broccoli V. Boncinelli E. Wurst W. (1999). The caudal limit of Otx2 expression positions the isthmic organizer. Nature 401, 164–168. 10.1038/43670 10490025

[B10] Bylund M. Andersson E. Novitch B. G. Muhr J. (2003). Vertebrate neurogenesis is counteracted by Sox1–3 activity. Nat. Neurosci. 6, 1162–1168. 10.1038/nn1131 14517545

[B11] Cadigan K. M. Waterman M. L. (2012). TCF/LEFs and Wnt signaling in the nucleus. Cold Spring Harb. Perspect. Biol. 4, a007906. 10.1101/cshperspect.a007906 23024173 PMC3536346

[B12] Cant N. B. Benson C. G. (2006). Organization of the inferior colliculus of the gerbil (*Meriones unguiculatus*): differences in distribution of projections from the cochlear nuclei and the superior olivary complex. J. Comp. Neurol. 495, 511–528. 10.1002/cne.20888 16498677 PMC2566545

[B13] Cavallaro M. Mariani J. Lancini C. Latorre E. Caccia R. Gullo F. (2008). Impaired generation of mature neurons by neural stem cells from hypomorphic Sox2 mutants. Development 135, 541–557. 10.1242/dev.010801 18171687

[B14] Chi C. L. Martinez S. Wurst W. Martin G. R. (2003). The isthmic organizer signal FGF8 is required for cell survival in the prospective midbrain and cerebellum. Development 130, 2633–2644. 10.1242/dev.00487 12736208

[B15] Cowan W. M. Martin A. H. Wenger E. (1968). Mitotic patterns in the optic tectum of the chick during normal development and after early removal of the optic vesicle. J. Exp. Zool. 169, 71–92. 10.1002/jez.1401690110 5696645

[B16] Dee A. Li K. Heng X. Guo Q. Li J. Y. H. (2016). Regulation of self-renewing neural progenitors by FGF/ERK signaling controls formation of the inferior colliculus. Development 143, 3661–3673. 10.1242/dev.138537 27578777 PMC5087642

[B17] Dessaud E. McMahon A. P. Briscoe J. (2008). Pattern formation in the vertebrate neural tube: a sonic hedgehog morphogen-regulated transcriptional network. Development 135, 2489–2503. 10.1242/dev.009324 18621990

[B18] Di Giovannantonio L. G. Di Salvio M. Omodei D. Prakash N. Wurst W. Pierani A. (2014). Otx2 cell-autonomously determines dorsal mesencephalon versus cerebellum fate independently of isthmic organizing activity. Development 141, 377–388. 10.1242/dev.102954 24335253

[B19] Edwards M. A. Caviness V. S. Schneider G. E. (1986). Development of cell and fiber lamination in the mouse superior colliculus. J. Comp. Neurol. 248, 395–409. 10.1002/cne.902480308 3722463

[B20] Graham V. Khudyakov J. Ellis P. Pevny L. (2003). SOX2 functions to maintain neural progenitor identity. Neuron 39, 749–765. 10.1016/S0896-6273(03)00497-5 12948443

[B21] Gray P. A. Hayes J. A. Ling G. Y. Llona I. Tupal S. Picardo M. C. D. (2010). Developmental origin of preBötzinger complex respiratory neurons. J. Neurosci. 30, 14883–14895. 10.1523/JNEUROSCI.4031-10.2010 21048147 PMC3056489

[B22] Guimera J. Weisenhorn D. V. Wurst W. (2006). Megane/Heslike is required for normal GABAergic differentiation in the mouse superior colliculus. Development 133, 3847–3857. 10.1242/dev.02557 16968817

[B23] Hagey D. W. Muhr J. (2014). Sox2 acts in a dose-dependent fashion to regulate proliferation of cortical progenitors. Cell. Rep. 9, 1908–1920. 10.1016/j.celrep.2014.11.013 25482558

[B24] Hesse K. Vaupel K. Kurt S. Buettner R. Kirfel J. Moser M. (2011). AP-2δ is a crucial transcriptional regulator of the posterior midbrain. PLoS One 6, e23483. 10.1371/journal.pone.0023483 21858141 PMC3153493

[B25] Joyner A. L. Liu A. Millet S. (2000). Otx2, Gbx2 and Fgf8 interact to position and maintain a mid-hindbrain organizer. Curr. Opin. Cell. Biol. 12, 736–741. 10.1016/s0955-0674(00)00161-7 11063941

[B26] Kala K. Haugas M. Lilleväli K. Guimera J. Wurst W. Salminen M. (2009). Gata2 is a tissue-specific post-mitotic selector gene for midbrain GABAergic neurons. Development 136, 253–262. 10.1242/dev.029900 19088086

[B27] Lahti L. Achim K. Partanen J. (2013). Molecular regulation of GABAergic neuron differentiation and diversity in the developing midbrain. Acta Physiol. (Oxf). 207, 616–627. 10.1111/apha.12062 23297792

[B28] Lakso M. Pichel J. G. Gorman J. R. Sauer B. Okamoto Y. Lee E. (1996). Efficient *in vivo* manipulation of mouse genomic sequences at the zygote stage. Proc. Natl. Acad. Sci. U. S. A. 93, 5860–5865. 10.1073/pnas.93.12.5860 8650183 PMC39152

[B29] Le Dréau G. Martí E. (2012). Dorsal-ventral patterning of the neural tube: a tale of three signals. Dev. Neurobiol. 72, 1471–1481. 10.1002/dneu.22015 22821665

[B30] Leung C. T. Coulombe P. A. Reed R. R. (2007). Contribution of olfactory neural stem cells to tissue maintenance and regeneration. Nat. Neurosci. 10, 720–726. 10.1038/nn1882 17468753

[B31] Li S. Xiang M. (2006). Barhl1 is required for maintenance of a large population of neurons in the zonal layer of the superior colliculus. Dev. Dyn. 235, 2260–2265. 10.1002/dvdy.20858 16752387 PMC2570113

[B32] Liu J. Xiao Q. Xiao J. Niu C. Li Y. Zhang X. (2022). Wnt/β-catenin signalling: function, biological mechanisms, and therapeutic opportunities. Signal Transduct. Target. Ther. 7, 3. 10.1038/s41392-021-00762-6 34980884 PMC8724284

[B33] Lu S. Bogarad L. D. Murtha M. T. Ruddle F. H. (1992). Expression pattern of a murine homeobox gene, Dbx, displays extreme spatial restriction in embryonic forebrain and spinal cord. Proc. Natl. Acad. Sci. U. S. A. 89, 8053–8057. 10.1073/pnas.89.17.8053 1355604 PMC49854

[B34] Martin D. M. Skidmore J. M. Philips S. T. Vieira C. Gage P. J. Condie B. G. (2004). PITX2 is required for normal development of neurons in the mouse subthalamic nucleus and midbrain. Dev. Biol. 267, 93–108. 10.1016/j.ydbio.2003.10.035 14975719

[B35] Masullo L. Mariotti L. Alexandre N. Freire-Pritchett P. Boulanger J. Tripodi M. (2019). Genetically defined functional modules for spatial orienting in the mouse superior colliculus. Curr. Biol. 29, 2892–2904. 10.1016/j.cub.2019.07.083 31474533 PMC6739420

[B36] Matsunaga E. Araki I. Nakamura H. (2001). Role of Pax3/7 in the tectum regionalization. Development 128, 4069–4077. 10.1242/dev.128.20.4069 11641229

[B37] McLaughlin T. O’Leary D. D. M. (2005). Molecular gradients and development of retinotopic maps. Annu. Rev. Neurosci. 28, 327–355. 10.1146/annurev.neuro.28.061604.135714 16022599

[B38] Miller M. W. Nowakowski R. S. (1988). Use of bromodeoxyuridine-immunohistochemistry to examine the proliferation, migration and time of origin of cells in the central nervous system. Brain Res. 457, 44–52. 10.1016/0006-8993(88)90055-8 3167568

[B39] Miyoshi G. Bessho Y. Yamada S. Kageyama R. (2004). Identification of a novel basic helix-loop-helix gene, Heslike, and its role in GABAergic neurogenesis. J. Neurosci. 24, 3672–3682. 10.1523/JNEUROSCI.5327-03.2004 15071116 PMC6729746

[B40] Nakatani T. Minaki Y. Kumai M. Ono Y. (2007). Helt determines GABAergic over glutamatergic neuronal fate by repressing Ngn genes in the developing mesencephalon. Development 134, 2783–2793. 10.1242/dev.02870 17611227

[B41] Okano H. J. Darnell R. B. (1997). A hierarchy of Hu RNA binding proteins in developing and adult neurons. J. Neurosci. 17, 3024–3037. 10.1523/JNEUROSCI.17-09-03024.1997 9096138 PMC6573636

[B42] Oliver D. L. Morest D. K. (1984). The central nucleus of the inferior colliculus in the cat. J. Comp. Neurol. 222, 237–264. 10.1002/cne.902220207 6699209

[B43] Peltopuro P. Kala K. Partanen J. (2010). Distinct requirements for Ascl1 in subpopulations of midbrain GABAergic neurons. Dev. Biol. 343, 63–70. 10.1016/j.ydbio.2010.04.015 20417196

[B44] Pierani A. Moran-Rivard L. Sunshine M. J. Littman D. R. Goulding M. Jessell T. M. (2001). Control of interneuron fate in the developing spinal cord by the progenitor homeodomain protein Dbx1. Neuron 29, 367–384. 10.1016/s0896-6273(01)00212-4 11239429

[B45] Puelles E. (2007). Genetic control of basal midbrain development. J. Neurosci. Res. 85, 3530–3534. 10.1002/jnr.21363 17549729

[B46] Puelles E. Acampora D. Lacroix E. Signore M. Annino A. Tuorto F. (2003). Otx dose-dependent integrated control of antero-posterior and dorso-ventral patterning of midbrain. Nat. Neurosci. 6, 453–460. 10.1038/nn1037 12652306

[B47] Sgaier S. K. Lao Z. Villanueva M. P. Berenshteyn F. Stephen D. Turnbull R. K. (2007). Genetic subdivision of the tectum and cerebellum into functionally related regions based on differential sensitivity to engrailed proteins. Development 134, 2325–2335. 10.1242/dev.000620 17537797 PMC2840613

[B48] Shamim H. Mahmood R. Logan C. Doherty P. Lumsden A. Mason I. (1999). Sequential roles for Fgf4, En1 and Fgf8 in specification and regionalisation of the midbrain. Development 126, 945–959. 10.1242/dev.126.5.945 9927596

[B49] Shoji H. Ito T. Wakamatsu Y. Hayasaka N. Ohsaki K. Oyanagi M. (1996). Regionalized expression of the Dbx family homeobox genes in the embryonic CNS of the mouse. Mech. Dev. 56, 25–39. 10.1016/0925-4773(96)00509-6 8798145

[B50] Sokolowski K. Esumi S. Hirata T. Kamal Y. Tran T. Lam A. (2015). Specification of select hypothalamic circuits and innate behaviors by the embryonic patterning gene dbx1. Neuron 86, 403–416. 10.1016/j.neuron.2015.03.022 25864637 PMC4484744

[B51] Szabo A. Dalmau J. Manley G. Rosenfeld M. Wong E. Henson J. (1991). HuD, a paraneoplastic encephalomyelitis antigen, contains RNA-binding domains and is homologous to Elav and Sex-lethal. Cell. 67, 325–333. 10.1016/0092-8674(91)90184-z 1655278

[B52] Tan S.-S. Valcanis H. Kalloniatis M. Harvey A. (2002). Cellular dispersion patterns and phenotypes in the developing mouse superior colliculus. Dev. Biol. 241, 117–131. 10.1006/dbio.2001.0505 11784099

[B53] Tran H.-N. Nguyen Q.-H. Jeong J. Loi D.-L. Nam Y. H. Kang T. H. (2023). The embryonic patterning gene Dbx1 governs the survival of the auditory midbrain via Tcf7l2-Ap2δ transcriptional cascade. Cell. Death Differ. 30, 1563–1574. 10.1038/s41418-023-01165-6 37081114 PMC10244374

[B54] Tran H.-N. Park W. Seong S. Jeong J.-E. Nguyen Q.-H. Yoon J. (2020). Tcf7l2 transcription factor is required for the maintenance, but not the initial specification, of the neurotransmitter identity in the caudal thalamus. Dev. Dyn. 249, 646–655. 10.1002/dvdy.146 31872525

[B55] Urbánek P. Wang Z. Q. Fetka I. Wagner E. F. Busslinger M. (1994). Complete block of early B cell differentiation and altered patterning of the posterior midbrain in mice lacking Pax5/BSAP. Cell 79, 901–912. 10.1016/0092-8674(94)90079-5 8001127

[B56] Waite M. R. Skidmore J. M. Billi A. C. Martin J. F. Martin D. M. (2011). GABAergic and glutamatergic identities of developing midbrain Pitx2 neurons. Dev. Dyn. 240, 333–346. 10.1002/dvdy.22532 21246650 PMC3079949

[B57] Wende C.-Z. Zoubaa S. Blak A. Echevarria D. Martinez S. Guillemot F. (2015). Hairy/Enhancer-of-Split MEGANE and proneural MASH1 factors cooperate synergistically in midbrain GABAergic neurogenesis. PLoS One 10, e0127681. 10.1371/journal.pone.0127681 25993409 PMC4439124

[B58] Wurst W. Bally-Cuif L. (2001). Neural plate patterning: upstream and downstream of the isthmic organizer. Nat. Rev. Neurosci. 2, 99–108. 10.1038/35053516 11253000

[B59] Zervas M. Blaess S. Joyner A. L. (2005). Classical embryological studies and modern genetic analysis of midbrain and cerebellum development. Curr. Top. Dev. Biol. 69, 101–138. 10.1016/S0070-2153(05)69005-9 16243598

