# Supplementary figures and images for: Dbx1 is a dorsal midbrain-specific determinant of GABAergic neuron fate and regulates differentiation of the dorsal midbrain into the inferior and superior colliculi
Source: Front Cell Dev Biol. 2024 Jan 26;12:1336308. doi: 10.3389/fcell.2024.1336308 (PMC10853453; doi:10.3389/fcell.2024.1336308)

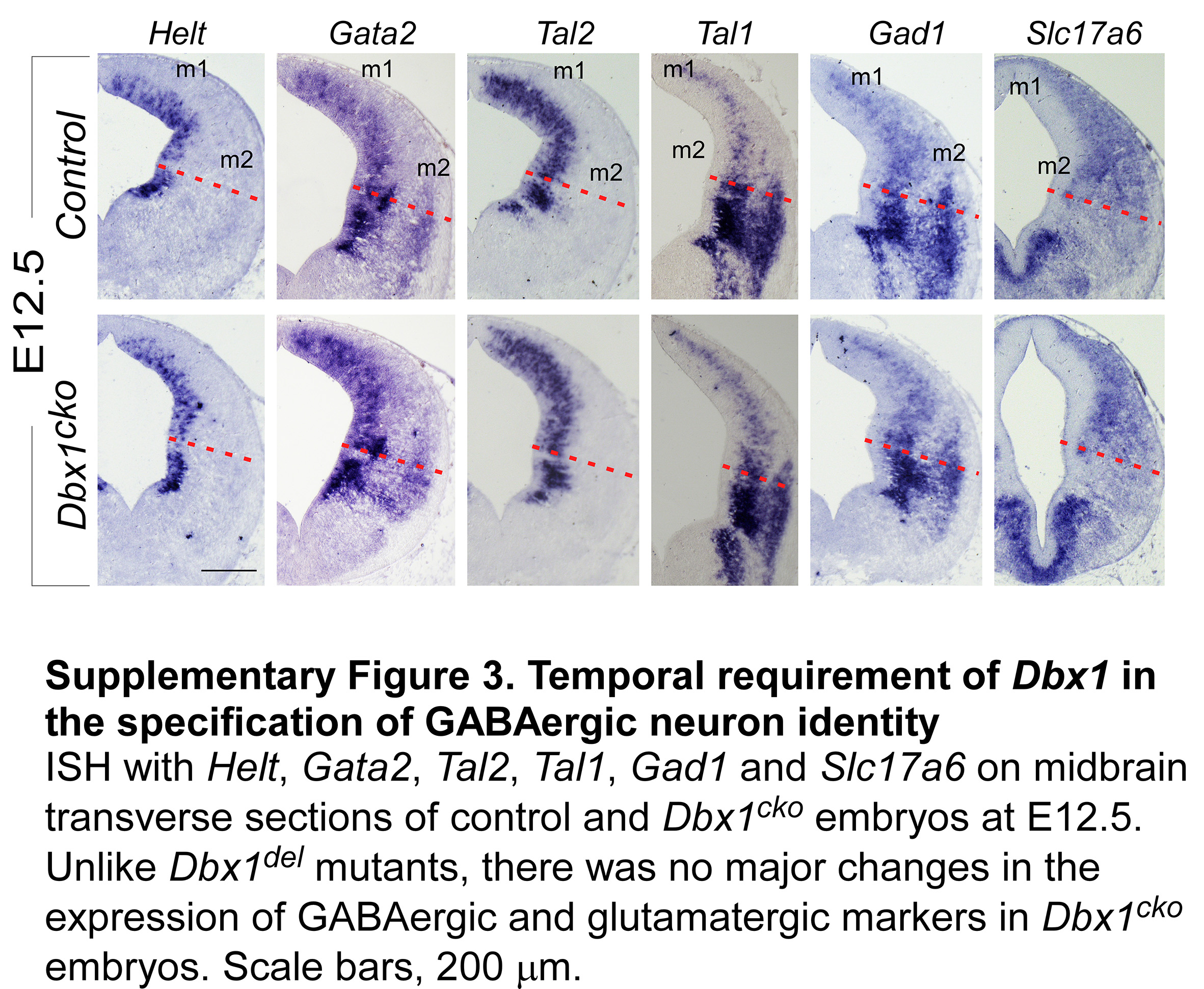

Supplement: Supplementary file 1 [file Image3.jpg]

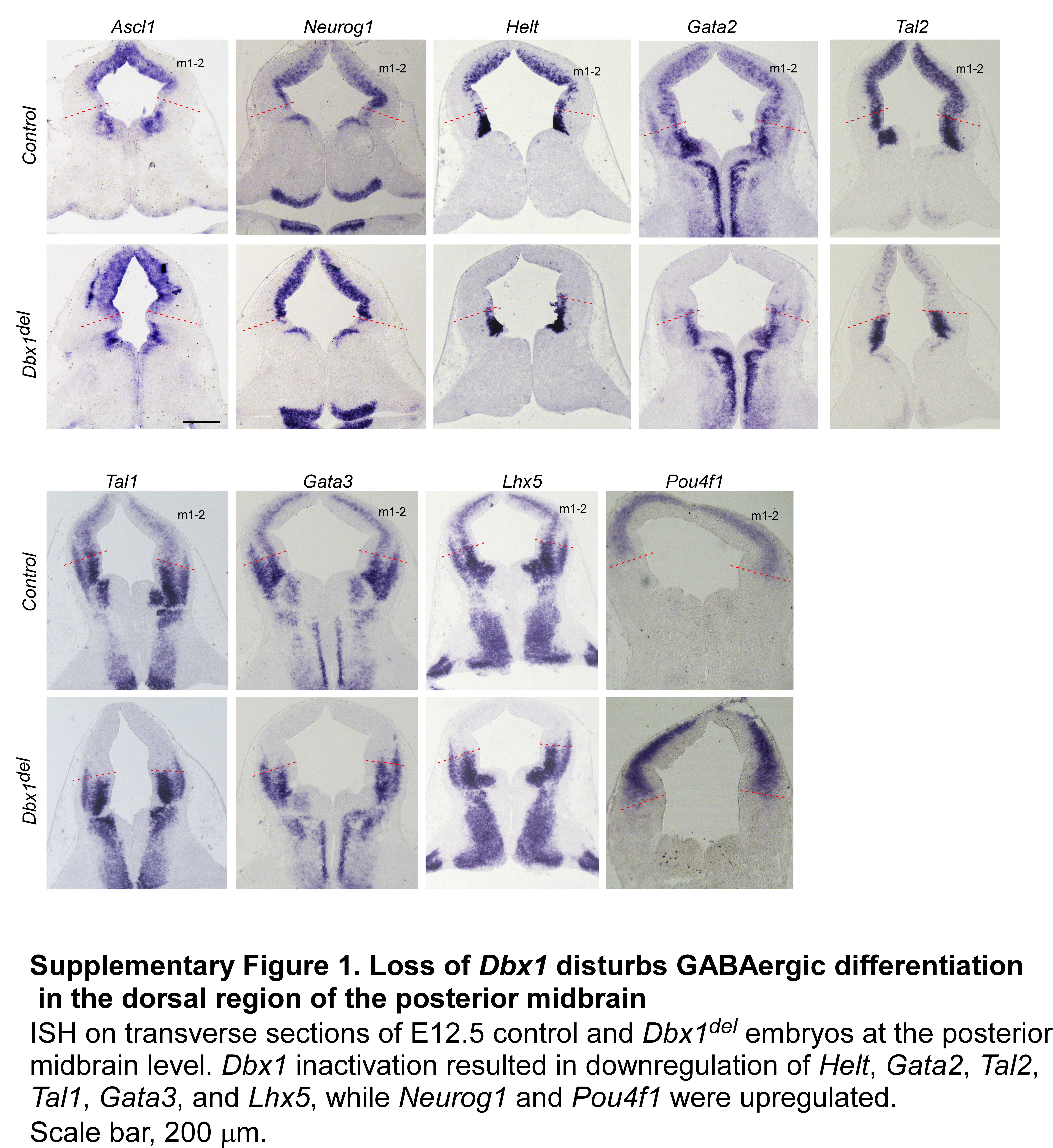

Supplement: Supplementary file 3 [file Image1.JPEG]

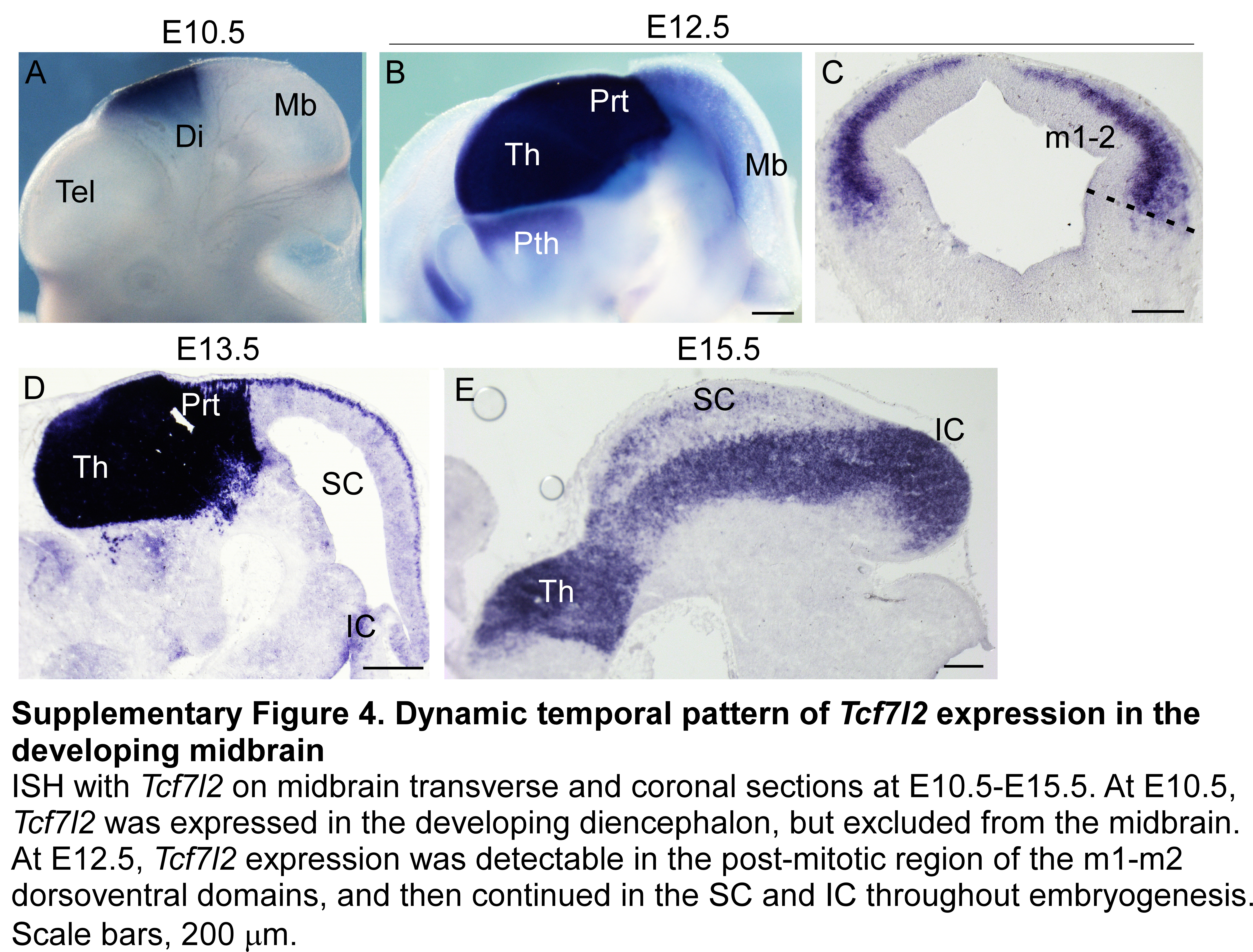

Supplement: Supplementary file 4 [file Image4.JPEG]

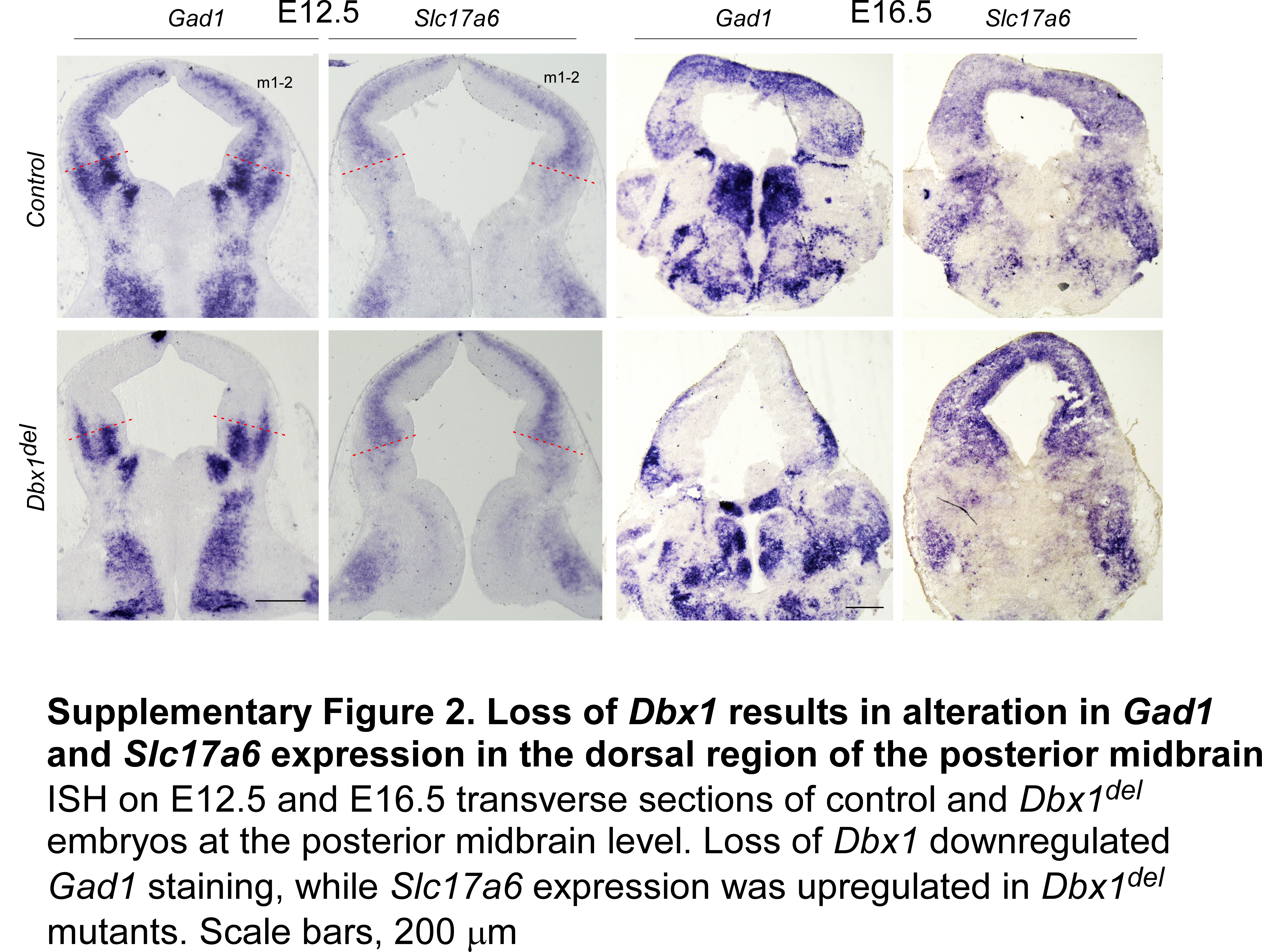

Supplement: Supplementary file 5 [file Image2.JPEG]

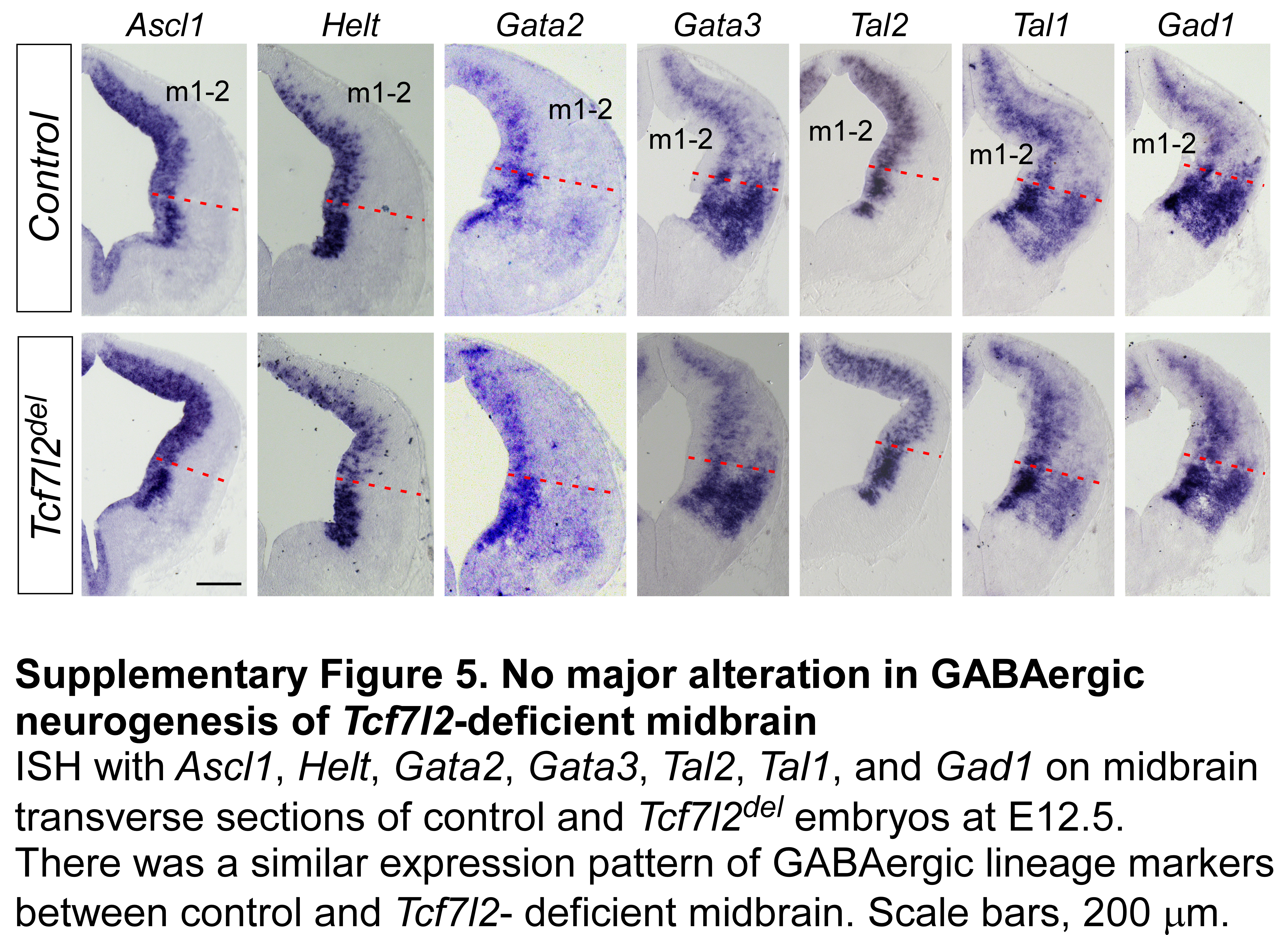

Supplement: Supplementary file 6 [file Image5.JPEG]
