# Supplementary material for: Dbx1 is a dorsal midbrain-specific determinant of GABAergic neuron fate and regulates differentiation of the dorsal midbrain into the inferior and superior colliculi
Source: Front Cell Dev Biol. 2024 Jan 26;12:1336308. doi: 10.3389/fcell.2024.1336308 (PMC10853453; doi:10.3389/fcell.2024.1336308)
Supplement: Supplementary file 2 [file Table1.DOCX]

Supplementary Table 1: RNA in situ Probes

| Gene | NCBI accession number | Nucleotides location |
| --- | --- | --- |
| Ap-2α | NM_011547 | 743-1363  (size: 621bp) |
| Ap-2δ | NM_001379074 | 1242-1560  (size: 319bp) |
| Ascl1 | NM_008553 | 1207-1672  (size: 466bp) |
| Barhl1 | NM_001164186 | 646-1110  (size: 465bp) |
| Dbx1 | NM_001005232 | 587-780  (size: 194bp) |
| Elavl3 | NM_010487 | 1196-1517  (size: 322bp) |
| Elavl4 | NM_010488 | 1250-1660  (size: 411bp) |
| Gad1 | AF326547 | 1791-2050  (size: 260bp) |
| Gata2 | NM_001355253 | 1625-2010  (size: 386bp) |
| Gata3 | NM_001417046 | 507-899  (size: 393bp) |
| Helt | DQ294234 | 166-997  (size: 832bp) |
| Lhx5 | U61155 | 1455-1760  (size: 286bp) |
| Mef2c | NM_025282 | 841-1027  (size: 187bp) |
| Meis2 | NM_010825 | 1372-1741  (size: 370bp) |
| Neurog1 | NM_010896 | 385-851  (size: 467bp) |
| Nkx2-2 | AH010986 | 4323-4875  (size: 553bp) |
| Nrgn | NM_022029 | 721-1196  (size: 476bp) |
| Ntng2 | NM_133500 | 443-881  (size: 439bp) |
| Otx2 | NM_144841 | 542-1087  (size: 546bp) |
| Pax7 | NM_011039 | 2634 - 3075  (size: 442bp) |
| Pitx2 | NM_001287048 | 1209-1584  (size: 376bp) |
| Pou4f1 | NM_011143 | 1593-2070  (size: 478bp) |
| Pou4f3 | NM_138945 | 208-570  (size: 363bp) |
| Slc17a6 | NM_080853 | 2445-3006  (size: 562bp) |
| Sox2 | NM_011443 | 889-1573  (size: 685bp) |
| Tal1 | NM_011527 | 775-1174  (size: 400bp) |
| Tal2 | NM_009317 | 105-520  (size: 416bp) |
| Tcf7l2 | NM_009333 | 1464-1624  (size: 161bp) |
